# Progress of selective catalytic reduction denitrification catalysts at wide temperature in carbon neutralization

**DOI:** 10.3389/fchem.2022.946133

**Published:** 2022-08-17

**Authors:** Dehai Lin, Longhui Zhang, Zilin Liu, Baodong Wang, Yifan Han

**Affiliations:** ^1^ National Institute of Clean and Low Carbon Energy, Beijing, China; ^2^ College of Chemical Esngineering, Zhengzhou University, Zhengzhou, Henan, China

**Keywords:** SCR denitration, wide- temperature catalyst, carbon neutralization, deactivation mechanism, regeneration

## Abstract

With the looming goal of carbon neutrality and increasingly stringent environmental protection policies, gas purification in coal-fired power plants is becoming more and more intense. To achieve the NOx emission standard when coal-fired power plants are operating at full load, wide-temperature denitrification catalysts that can operate for a long time in the range of 260–420°C are worthy of study. This review focuses on the research progress and deactivation mechanism of selective catalytic reduction (SCR) denitration catalysts applied to a wide temperature range. With the increasing application of SCR catalysts, it also means that a large amount of spent catalysts is generated every year due to deactivation. Therefore, it is necessary to recycle the wide temperature SCR denitration catalyst. The challenges faced by wide-temperature SCR denitration catalysts are summarized by comparing their regeneration processes. Finally, its future development is prospected.

## Introduction

**FIGURE 1:**
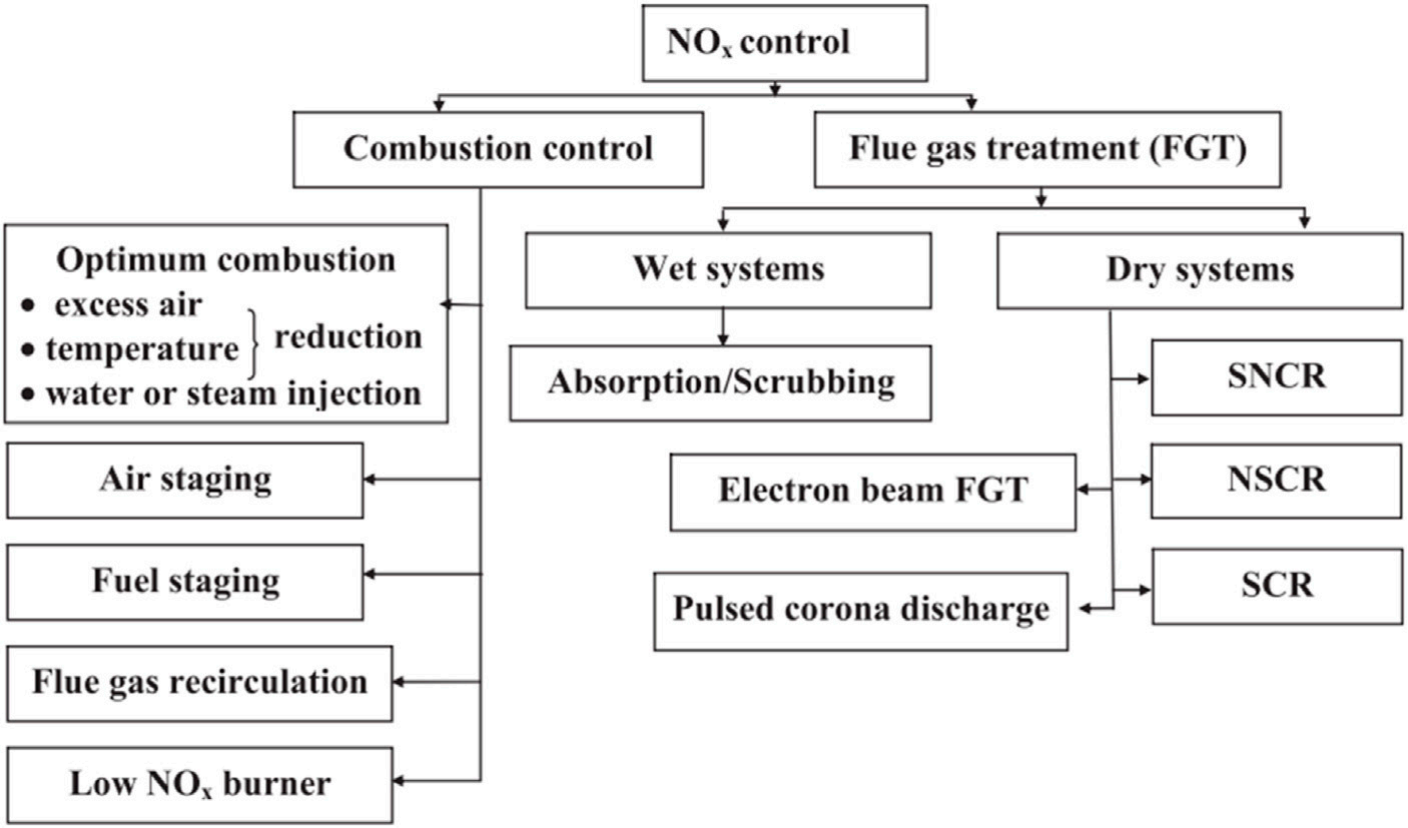
Overview of NO_X_ removal technology (Mladenović et al., 2018).

With the entry into force of the Paris Agreement, the carbon reduction situation is grim. The carbon emission of the power industry accounts for nearly half of the total emissions, and the coal-fired power industry will become the main target of carbon dioxide emission reduction (Mallapaty, 2020; Jia and Lin, 2021; Williams et al., 2021). Conventional air pollutants are no longer a binding factor for the development of coal power, and carbon emission reduction will become an important limiting factor. Coal power companies will choose cleaner and low-carbon power generation technologies. Electric power companies will greatly increase the installed capacity of new energy, but such power points have the characteristics of intermittent, fluctuating, anti-peak regulation, and low prediction accuracy and capacity reliability. Therefore, a high proportion of new energy sources are connected to the power grid, which makes the deep peak-shaving and low-load operation of coal-fired power plants the norm (Xue et al., 2019; Du et al., 2021; Wang et al., 2021). As shown in Figure 1, considering that the combustion of fossil fuels such as coal, oil, and natural gas is the main source of NO_X_ emissions, emission control around NOx will be a long-term task (Mirhashemi and Sadrnia, 2020; Cai et al., 2021).

The denitration devices are generally put into operation in active coal-fired power plants, and the main denitration technology is to install SCR denitration between the economizer and the air preheater. SCR denitration technology is a denitration technology that selectively converts NO_X_ in flue gas into N_2_ and water by using reducing agent (NH_3_ or urea, etc.) under appropriate temperature and catalyst conditions. Selective Catalytic Reduction (SCR) NO_X_ is one of the most effective NO_X_ removal technologies available (Zhang et al., 2016; Andana et al., 2021; Zhang et al., 2022). Widely used in NO_X_ control of coal-fired power plants, industrial furnaces (stationary sources), diesel vehicles and ocean-going ships (mobile sources). Its basic principle is to use ammonia as a reducing agent, and under the action of a catalyst, NOx in the flue gas can be selectively reduced to N_2_ and H_2_O. The core of SCR technology is the SCR catalyst. The current commercial SCR catalysts are mainly composed of V_2_O_5_-WO_3_(MoO_3_)/TiO_2_, which exhibit excellent NOx conversion and good poisoning resistance in the range of 300–420°C (Zheng et al., 2019; Shen et al., 2021a; Wu et al., 2021; Guo et al., 2022). This operating temperature range is similar to the exhaust gas temperature of coal-fired power plants. Therefore, since its first application in the 1970s, SCR technology has been the first to be applied on a large scale in coal-fired power plants. However, there are still some problems in the use of V-based catalysts. For example, V_2_O_5_ is very easy to sublime, and it will produce huge biological toxicity after entering the external environment. The temperature operating window of the catalyst is relatively narrow, a large amount of N_2_O is generated at high temperature, and the catalyst has poor high temperature stability. In addition, in order to meet the working temperature of the catalyst, the SCR catalytic unit is generally installed before the discharge flue gas electrostatic precipitator and desulfurization device of the coal-fired power plant. Therefore, the catalyst will gradually be poisoned by alkali/alkaline earth metals, phosphorus, heavy metals and fly ash in the flue gas (Yan et al., 2019; Xu D. et al., 2020; Xu L. et al., 2020; Lisi and Cimino, 2020; Miao et al., 2020). To solve the above problems, the SCR catalyst is moved after the dust removal and desulfurization device, but at the same time, the flue gas temperature will drop below 250°C, which requires the SCR catalyst to have good denitration efficiency at low temperature. In addition, there are many industrial kilns (steel, cement, glass) whose exhaust emissions are significantly lower than coal-fired power plants, further making the development of low-temperature SCR catalysts with excellent denitration performance an urgent need to control exhaust gas and improve the atmospheric environment.

To cope with the transformation of carbon neutral enterprises, the proportion of new energy installed capacity is increasing, and new problems and difficulties are coming. On the one hand, the installed capacity of thermal power shows a new normal of local excess and the number of utilization hours decreases year by year; on the other hand, the randomness and volatility of wind and solar power generation are large, and large-scale consumption is a major challenge to the comprehensive adjustment capacity of the power grid system. Therefore, flexible power supply will become a scarce resource. Coal-fired power has become the main force involved in peak regulation, frequency regulation and voltage regulation of the power system through flexible technological transformation. In order to encourage thermal power units to meet flexible peak shaving capabilities through technological transformation, various regional power grids have promulgated electric power auxiliary market operation rules of different standards, which have significantly improved the incentives for thermal power units to participate in deep peak shaving (Gu et al., 2016; Xu and Shen, 2021). The unit deviates from the original design conditions during low-load operation, and the flue gas parameters are quite different from those during normal high-load operation, which adversely affects the operation of various equipment in coal-fired power plants. For example, increasing the actual operating coal consumption of the unit, affecting the cooling of the boiler, reducing the NO_X_ control ability of the denitrification system, and increasing the amount of ammonia injection and the generation of ammonium hydrogen sulfate (Zhang Y. et al., 2019; Shen and Zhang, 2020; Jiao et al., 2021). This not only significantly increases the operating cost of the unit, but also poses a greater safety risk to the operation of the boiler, catalyst, air preheater and electrostatic precipitator system. In-depth peak regulation and ultra-low emission of pollutants have certain technical contradictions. In addition to the low-load boiler combustion stabilization technology and thermal electrolysis coupling, the wide-load operation transformation of denitrification facilities is also one of the key tasks in the flexibility transformation of thermal power units (Lin et al., 2019). The flue gas temperature is lower than the minimum temperature of 300 °C (Zhang et al., 2020) for the safe operation of traditional V_2_O_5_/WO_3_-TiO_2_ catalysts. The new situation of low-load deep peak shaving of coal-fired units and low-temperature denitrification in non-power industries requires the development of new wide-temperature denitrification catalysts and corresponding processes to meet the latest national pollutant emission standards.

This review focuses on the research progress of SCR catalysts at wide temperature. Firstly, the current research status of SCR out-of-stock technology is summarized. Then, according to the clear out-of-stock mechanism, combined with the application temperature range of SCR catalyst, the challenges faced by the development and application of SCR catalyst under wide temperature are discussed. Combined with the poisoning mechanism and regeneration technology of SCR catalysts, constructive suggestions for the design and development of SCR catalysts for wide temperature are put forward.

## SCR technology

### SCR denitration mechanism

**FIGURE 2:**
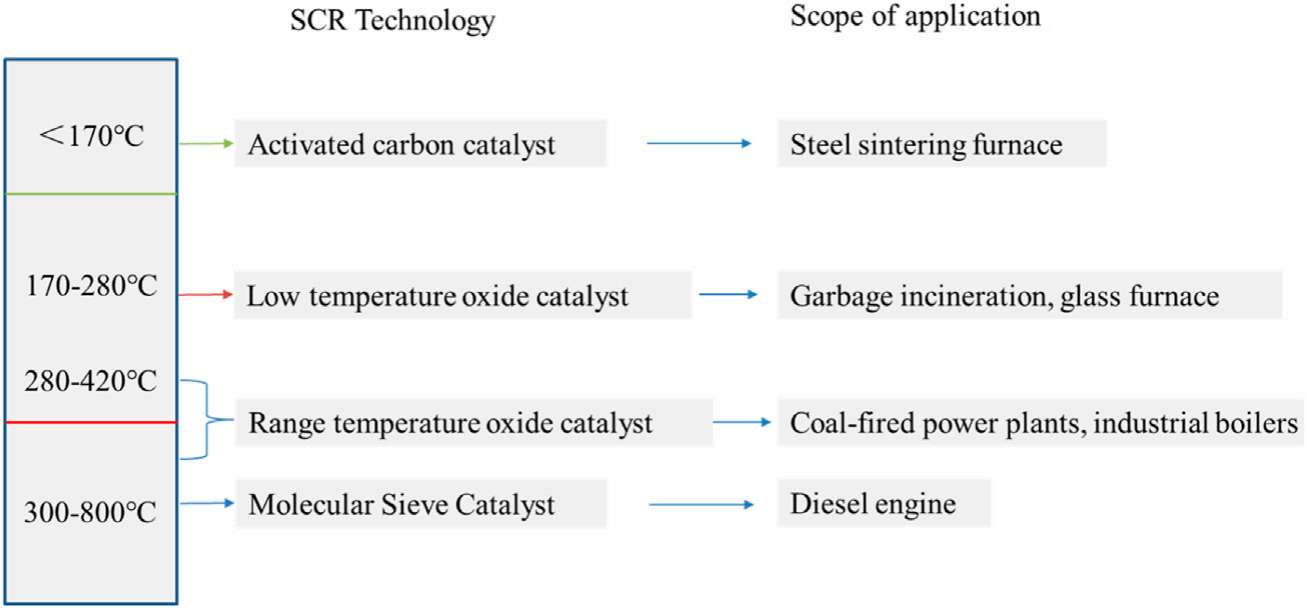
SCR technology route for controlling nitrogen oxide emissions.

NH_3_ selective catalytic reduction or SCR refers to the process of selectively generating N_2_ and H_2_O through the redox reaction of NO_X_ and reducing gas NH_3_. The reaction can be carried out under both aerobic and anaerobic conditions to obtain the same product. The technical route of SCR to control nitrogen oxide emissions is shown in Figure 2. The reaction temperature of this technology is generally in the range of 280–450°C, and the specific reaction formula is as follows (Damma et al., 2019):

$$4NO+4NH_{3}+O_{2}\to 4N_{2}+6H_{2}O$$ (1)

$$6NO_{2}+8NH_{3}\to 7N_{2}+12H_{2}O$$ (2)

$$2NO+4NH_{3}+2O_{2}\to 3N_{2}+6H_{2}O$$ (3)

$$6NO+4NH_{3}\to 5N_{2}+6H_{2}O$$ (4)

$$2NO+2NO_{2}+4NH_{3}\to 4N_{2}+6H_{2}O$$ (5)

$$2NO_{2}+4NH_{3}+O_{2}\to 3N_{2}+6H_{2}O$$ (6)

Without catalysis, the reactions of Eqs 1, 2 can only occur in a narrow high temperature range (900–1,000°C). After using a suitable catalyst, the activation energy of the reaction decreases, and the reaction temperature drops to 300–400°C, which is close to the exhaust gas temperature between the boiler economizer and the air preheater, and is suitable for the actual working conditions of the power plant. In the actual flue gas denitrification process, since NO accounts for more than 95% of the total NO_X_, Eq. 1 is a typical SCR reaction formula. When there is no O_2_ or the O_2_ content is low, it is mainly carried out according to Eq. 4. When the NO_2_/NO ratio in the flue gas is about 1, the reaction rate of Eq. 5 is higher, which is the main reaction step (Koebel et al., 2001) of the SCR reaction. When the O_2_/NO ratio in the flue gas is greater than 1, Eqs 3, 6 are the main reaction steps, but the rate is relatively low. In actual flue gas, O_2_ generally exists. Therefore, as the reaction temperature increases (above 300 °C), NH_3_ will react with O_2_, that is, a side reaction will occur, generating N_2_, NO, N_2_O, etc., and NH3 will also be pyrolyzed to generate N_2_ and H_2_. There are mainly three types of side reactions that threaten the performance and operation safety of the SCR system, namely the oxidation and decomposition of NHs, the oxidation of SO_2_ and the formation of ammonium salts (NH_4_HSO_4_, (NH_4_)_2_SO_4_). Part of the ammonia will be oxidized, To generate N_2_, NO and other nitrogen-containing substances, the possible reactions are (Damma et al., 2019):

$$4NH_{3}+3O_{2}\to 2N_{2}+6H_{2}O$$ (7)

$$4NH_{3}+5O_{2}\to 4NO+6H_{2}O$$ (8)

$$2NH_{3}\to N_{2}+3H_{2}$$ (9)

At 350°C, the reaction in which NH3 in Eq. 8 is oxidized to NO and the reaction in Eq. 9 that NH_3_ is directly decomposed into N_2_ can proceed, and the reaction above 450°C is more intense. In industrial applications, the temperature is controlled at 400°C, and the side reaction is mostly the oxidation of NH_3_ to N_2_, which is the reaction Eq. 7. The oxidation of ammonia not only increases the ammonia supply rate required for denitration, but also reduces the oxygen adsorbed on the inner surface of the catalyst, which may lead to insufficient catalyst volume. It is necessary to prevent the oxidation of ammonia during operation.

### The main influencing factors of SCR

There are many factors that affect the SCR denitration system, and the core lies in the catalyst. The structure, type and surface area of the catalyst have a great influence on the performance of the catalyst. Other important operating parameters, including flue gas temperature, space velocity, ammonia-nitrogen ratio, and oxygen concentration, also have a significant impact on catalyst activity.

The space velocity ratio refers to the ratio of the volume (mass) of the catalyst per unit time to the volume of the reaction gas flowing through the catalyst under standard pressure and temperature. It is a measure of the residence time of the flue gas on a unit volume of catalyst and determines to a certain extent whether the reactants are completely reacted. Increasing the space velocity ratio means that the residence time of flue gas in the reactor is shortened, the reaction may be incomplete, and the flow rate of flue gas increases, and the amount of ammonia escape increases. Reducing the space velocity ratio means that the residence time of the flue gas in the reactor is prolonged, and the removal rate of nitrogen oxides will increase. But the amount of catalyst increases and the cost increases. Therefore, a moderate airspeed ratio should be selected, which not only improves the denitration efficiency, but also reduces the economic cost.

The flue gas temperature affects the denitration activity of the SCR catalyst. The catalyst type and flue gas composition directly determine the optimum temperature for the denitration reaction. The temperature range for most commercial catalysts today is 250–420°C. When the flue gas temperature is close to the optimum temperature, the catalyst has the highest denitration activity and the reaction rate increases. The same denitration effect only needs a smaller amount of catalyst, thereby saving the cost of the SCR denitration system, which is beneficial to the operation and performance of the SCR denitration system.

According to the SCR fast reaction equation, theoretically, in the reaction of equal amounts of NH_3_ and NO, H_2_O and N_2_ can achieve 100% conversion. The typical ratio of ammonia to nitrogen is 1.05. Excessive NH3 will produce ammonia escape phenomenon and cause secondary pollution, but the theoretical denitrification efficiency cannot be achieved when the amount of NH_3_ is insufficient. Oxygen concentration: O_2_ in the flue gas is very important in the SCR reaction and can improve the reaction rate. Studies have shown that the oxidation of NO and O_2_ on the catalyst surface is a crucial link in the SCR reaction process. In the SCR reaction, NO is first dissociated to generate O atoms, and then the catalyst active sites are reduced by the generated 0 atoms. The O_2_ in the flue gas will regenerate the active sites of the catalyst, so that the SCR reaction can be carried out. The reaction of NO with NHs is very slow under anaerobic conditions due to the weak oxidizing ability of NO on the catalyst surface.

### Arrangement of SCR reactor

Generally, the SCR denitration reaction device is arranged before the air preheater, and about 95% of the coal-fired power plants adopt this installation. The advantage of this installation is that the flue gas temperature can meet the requirements of the actual working conditions, but the flue gas contains a large amount of fly ash and impurities, which will block the catalyst pores and affect the normal progress of the SCR denitration reaction. The cost of the catalyst accounts for about one-third of the cost of the overall denitration device, so adopting a suitable, low-cost, and economical arrangement is the key to the SCR denitration reaction. Appropriate arrangements need to be taken according to the type of fuel, combustion, etc. There are generally three arrangements:

(1) High temperature and high dust arrangement: This arrangement is to place the SCR denitration reactor before the air preheater. At this time, the temperature region just reaches the active temperature window of the denitration catalyst, and there is no need for a secondary heating process, which saves the actual operating cost of the power plant. However, the high temperature flue gas contains a large amount of components harmful to the catalyst, such as fly ash, impurities and SO_2_, and the working conditions of this reaction are relatively “bad".
(2) High temperature and low dust arrangement: This arrangement is to place the SCR denitration reactor between the electrostatic precipitator and the air preheater. The advantage of this arrangement is that after the flue gas passes through the dust removal device, the fly ash and impurities in the flue gas will be effectively removed. The temperature range for this reaction is about 300–400°C, which avoids the problem of catalyst deactivation caused by fly ash. However, excessively high flue gas temperature will still lead to sintering of the catalyst.
(3) Low-temperature and low-dust arrangement: In this arrangement, the SCR denitration reactor is placed after the dust removal device and the flue gas desulfurization device. After the flue gas is dedusted and desulfurized, fly ash, impurities and SO_2_ will be effectively removed. The working conditions become relatively “clean”, and the degree of damage to the denitration catalyst is relatively small. This helps extend the life of the catalyst and saves the actual operating costs of coal-fired power plants.

## Research on SCR denitration catalyst at wide temperature

Denitrification technology emerged and developed with the increasingly severe environmental situation caused by the increasing emission of nitrogen oxides. Excessive emissions of nitrogen oxides are mainly concentrated in mobile sources such as vehicle exhaust emissions and stationary sources such as thermal power plants and chemical plants. two sources. Selective catalytic reduction technology is currently the most commonly used, most efficient and most economical denitration technology, and its core is the catalyst (Gao et al., 2017; Wang et al., 2017). The denitration catalyst is an important part of the whole SCR reaction process. For NO_X_ emitted from stationary sources, since the combustion temperature is generally concentrated between 300 and 400°C, it is necessary to find medium and high temperature denitration catalysts. Generally, industrial kilns have a large production scale and a large amount of flue gas emissions. In order to achieve the purification effect, the amount of catalyst is large, and the requirement for space velocity is relatively low. Moreover, the industrial furnace flue gas purification device occupies a relatively large space, and the catalyst replacement is relatively easy, so the catalyst with low cost and high performance is the best choice. For diesel vehicles in mobile sources, due to higher fuel economy and lower CO_2_ emission requirements, the exhaust temperature of diesel vehicles will be further reduced, so there is an extremely urgent need to improve the low-temperature activity of catalysts. On the other hand, the regular regeneration of the particulate filter in the diesel vehicle exhaust after-treatment device will generate a high temperature above 800°C, which puts forward higher requirements for the hydrothermal stability of the SCR catalyst. In addition, due to its space effect and the difficulty of catalyst replacement, the catalyst must withstand high space velocity and long service life, and the requirements for the catalyst are relatively high. It can be seen that different SCR catalysts need to be found for different NO_X_ pollution sources. SCR catalysts are divided into temperature ranges (Fu-Dong et al., 2011), generally divided into medium and low temperature SCR denitration catalysts and high temperature SCR denitration catalysts. The composition of SCR catalyst includes three parts, active component, carrier and promoter. There is a certain relationship between the properties of the catalyst and the synergy between the carrier and the active components, and the selection of additives can improve some deficiencies of the original catalyst, better disperse the active components, and improve the overall denitration performance of the catalyst. The current research on commonly used low-temperature SCR catalysts is divided into the following categories: metal oxide catalysts, carbon-based catalysts, noble metal catalysts, and molecular sieve catalysts (Ruliang et al., 2019; Ma H. et al., 2021). High temperature SCR catalysts are divided into metal oxide catalysts and molecular sieve catalysts.

### Research on high- temperature SCR denitration catalyst

High-temperature SCR denitration catalysts are mainly used in a series of equipment that produces a large amount of high-temperature flue gas, such as fuel engines and waste incinerators. The reaction temperature of high-temperature catalysts is above 500°C, and the crystal lattice of commonly used vanadium-titanium system catalysts is destroyed under high temperature conditions, resulting in a decrease in catalytic activity. However, the common high-temperature SCR denitration catalyst carrier selection conditions require higher thermal stability at lower temperature, and molecular sieve catalysts and metal oxides are usually selected as carriers for loading (Zhigang et al., 2010; Albert et al., 2019; Shen et al., 2021b).

Xu et al. (Xu et al., 2019) studied the effect of the addition of C_3_H_6_ on the wide temperature range of high temperature catalysts by studying different types of molecular sieves including ZSM-5, SSZ-13, SAPO-34 and SBA series molecular sieves, supported active components Cu and Fe. The wide temperature range of the high temperature SCR catalyst was found by the characterization of H2-TPR, XRD and C_3_H_6_-TPD. Under the action of the auxiliary C_3_H_6_, the dispersion of the active components and the pore size of the molecular sieve carrier can be changed, which will be beneficial to the continuation of a wide temperature range with high activity. The metal oxide V_2_O_5_ is usually used as the active component in low-temperature SCR denitration catalysts, and due to its high dispersion on the surface of TiO_2_, it becomes an industrialized low-temperature SCR denitration catalyst with high activity. Due to the specific oxidation potential of V_2_O_5_, the NH_3_ adsorbed by the carrier and gaseous NO can be directly activated to generate N_2_. At high temperature, TiO_2_ will transfer from anatase to rutile, affecting the crystal structure, resulting in a decrease in activity. Tran et al. (Tran et al., 2017) changed the phase transition temperature of the catalyst support by adding WO_3_ to the V_2_O_5_/TiO_2_ and V_2_O_5_/SiO_2_ catalysts, and confirmed that the addition of WO_3_ formed the defect of Ti-O bonds by a series of characterizations such as XRD and IR. A higher specific surface area was obtained, the conversion temperature of rutile TiO_2_ was increased, and a new high-temperature catalyst with thermal stability was formed.

### Research on medium- and low- temperature SCR denitration catalysts

Medium and low temperature SCR denitration catalysts are industrially applied to the tail of industrial furnaces or boilers. They have the following characteristics (Park et al., 2013): Reduce the poisoning effect of the trace elements K, Ca, As, etc. Reduced catalyst deactivation caused by SO_2_ poisoning. It is located behind the dust removal device, which solves the problem of pore blockage caused by too many impurities in the catalyst, increases the service life of the catalyst, and reduces the operating cost. The medium and low temperature SCR catalyst device is arranged on the ground, which is less affected by the pipeline, and the temperature resistance of the reaction equipment is low, which has little impact on the related devices.

At present, most of the research on medium and low temperature SCR denitration catalysts by scholars from all walks of life is the research on active components and carriers. On the basis of the medium temperature catalyst, the temperature window is widened, in order to meet the situation that the flue gas temperature cannot reach the design temperature due to the reduced load operation in the actual operation of the power plant. The active components of the medium and low temperature SCR catalysts are usually (Kröcher and Brandenberger, 2012) metal manganese oxides, copper-based catalysts, iron-based catalysts, chromium oxide and nickel oxide and other metal oxides, which are composed of one or more single-metal or bi-metal catalysts. The supports selected in the research process of medium and low temperature SCR denitration catalysts include (Li, 2009; Qiu et al., 2015; Zhang M. et al., 2019) molecular sieve, TiO_2_, Al_2_O_3_, carbon materials, etc. New low-temperature SCR denitration catalytic material with high NOx conversion rate.

(1) Precious metal catalysts

Early research on SCR catalysts mostly focused on precious metals, using precious metals such as Pt, Rh, and Pd as active components, and granular Al_2_O_3_ and monolithic ceramics as carriers (Cavataio et al., 2009), which are still used in diesel engine denitration technology. If C3H3 is used as reducing agent, the honeycomb catalyst Pt/Al_2_O_3_ has good denitration activity (Kim et al., 2012). The d electron orbital of precious metals is not filled, so it can enhance the adsorption of reactants and has high denitration performance, but because of its strong oxidizing property, it has a strong oxidizing effect on reducing agents, resulting in additional side effects and consuming a large amount of reduction. The cost of the agent further increases, and the active temperature window is narrow, the selectivity is poor, the sulfur resistance is very poor, and it is very easy to be poisoned and inactivated. If a certain amount of Ag is doped into the silver-titanium catalyst, the denitration efficiency can reach 70% at 250°C, but when the temperature increases, NH3 is deeply oxidized by Ag to NO_2_(Lee et al., 2021). Due to the high cost, noble metal catalysts are gradually replaced by metal oxide catalysts with the development of SCR catalysts.

(2) Metal oxide catalysts

Metal oxide catalysts use metal oxides as active components, and are divided into supported and unsupported types. Supported metal oxide catalysts are widely used. Among them, TiO_2_ is the most common carrier, which is generally anatase structure, which can improve the dispersion of active components, thereby obtaining good catalytic activity; the carrier TiO_2_, to a certain extent, is responsible for the reaction between the SO_3_ generated by the oxidation of SO_2_ and the carrier. In addition, the sulfate formed on the carrier TiO2 is unstable and easy to decompose, so the active sites on the carrier TiO_2_ are not easily covered by sulfate, thereby improving the sulfur resistance and stability of the catalyst. Composite carriers such as TiO_2_-SiO_2_(Peng et al., 2017), TiO_2_-ZrO_2_(Fan et al., 2018), TiO_2_-SnO_2_(Patil et al., 2018), TiO_2_-Al_2_O_3_(Wang et al., 2018), etc. have also been extensively studied. For example, TiO_2_-SnO_2_(Ming’e et al., 2016; Sönmezoğlu et al., 2011) carriers have large specific surface area, excellent denitration activity and anti-sulfur and water-resistance.

The active components are mostly transition metal oxides. Most of these catalysts are non-stoichiometric compounds, which have the characteristics of positive and negative ion vacancy and lattice oxygen, and have specific active centers, which can promote redox reactions. At present, the most widely used SCR denitration catalysts are V_2_O_5_/TiO_2_ series catalysts, which are doped with one or both of WO_3_ or MoO_3_ as additives to improve the denitration activity and sulfur resistance at medium and high temperature (Casanova et al., 2006; Guo et al., 2009). V_2_O_5_ is the main active component of the catalyst, which can provide more acid sites and has good denitration activity at high temperature. However, it is easy to oxidize SO_2_ in the flue gas to form SO3, resulting in the deactivation of the catalyst. In the process of SCR denitration, the conversion rate of SO_2_/SO_3_ should be controlled within 1%, so the loading of V_2_O_5_ in the catalyst should be controlled. V_2_O_5_ has carcinogenic effects, and the recycling of waste catalysts is not mature at present. V_2_O_5_ is harmful to the environment and human health, so it is not suitable to be used in large quantities.

**FIGURE 3:**
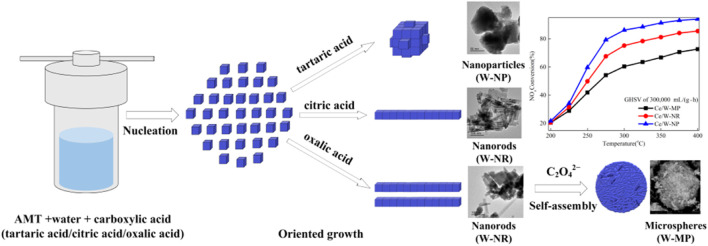
Preparation and performance of WO_3_ supported CeO_2_ SCR catalyst (He et al., 2021).

At present, the research hotspots of transition metal oxide catalysts mainly focus on metal oxides such as Ce-based, Mn-based and Fe-based. CeO_2_ is favored by many researchers due to its good oxygen storage capacity and the price change of Ce^3+^/Ce^4+^. In addition to the aforementioned CeO_2_ as a co-agent to modify the SCR performance of commercial V_2_O_5_-WO_3_/TiO_2_, CeO_2_ itself is often used as a support or active component of NH_3_-SCR catalysts. When CeO_2_ is used as a carrier, it can not only effectively disperse the active species, but also adjust the interaction between CeO_2_ and the surface of the active components through the electronic effect, and improve the SCR performance of the catalyst. For example, He et al. (He et al., 2021) supported WO_3_ with different morphologies on CeO_2_ by the method of isovolumetric impregnation (as shown in Figure 3), and the results showed that due to the interaction of W ions and Ce ions, a new Ce^4+^+W^5+^ The formation of Ce^3+^+W^6+^ redox cycle greatly promotes the redox performance of the catalyst. Among them, compared with nanorods (W-NR) and nanorod self-assembled microspheres (W-MP), WO_3_ nanoparticles (W-NP) have the best redox ability, and its W^5+^/(W^5+^+W^6+^) The molar ratio of α is the largest, and the concentration of Ce^4+^ on the surface of Ce/W-NPs is the highest, which exhibits the best redox performance, thereby promoting the SCR performance. When CeO_2_ is used as the active component of SCR catalyst, it is easy to deactivate due to the instability of pure CeO_2_ at high temperature (Ma S. et al., 2021). Therefore, many researchers tried to add different transition metals into CeO_2_ to improve its stability and SCR activity. For example, Liu et al. (Liu et al., 2014) added Mo atoms to CeTi catalysts by co-precipitation to enhance the acidity of the catalysts, so that the catalysts exhibited not only good SCR performance but also good water and sulfur resistance. Similar doping components also include Cu(Gao et al., 2010a), Mn (Jin et al., 2010), W (Jin et al., 2010), Nb(Qu et al., 2013), Ti (Liu Z. et al., 2018), etc.

For Ce-based composite metal oxides formed by doping transition metal oxides, the determination of active sites is controversial. For example, He et al. (Xu et al., 2008) impregnated TiO_2_ supports with active components with different CeO_2_ contents by impregnation, showing excellent SCR performance and N_2_ selectivity. Some researchers believe that the highly dispersed CeO_2_ in the Ce-Ti catalyst is the active site (Xu et al., 2008; Gao et al., 2010b), but the characterization results did not detect the existence of CeO_2_ crystal phase. Later, Li et al. (Li et al., 2012) discovered for the first time that the amorphous Ce-O-Ti short-range ordered structure was the active site of the Ce-Ti composite oxide catalyst by XRD, high-power transmission electron microscopy (HRTEM) and XAFS. The determination of metal oxide active sites provides a new technology and lays the foundation for the construction of efficient SCR catalysts.

**FIGURE 4:**
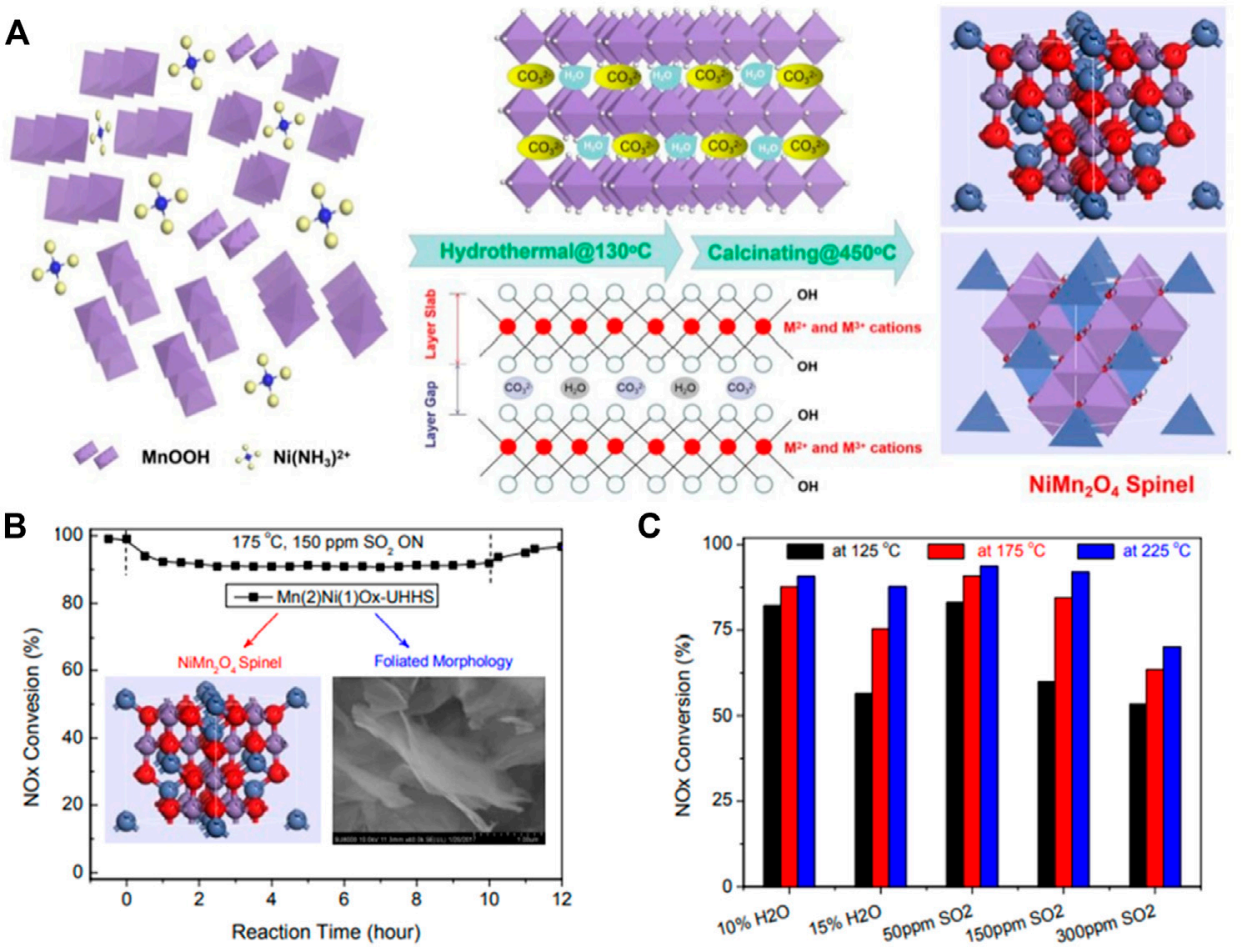
Novel Mn-Ni spinel nanosheets fabricated by UH and UHHS and their NOx removal, H_2_O and SO_2_ resistance (Gao et al., 2020).

Mn-based catalysts are considered as one of the most promising catalysts due to their excellent low-temperature NH_3_-SCR performance (Liu J. et al., 2018). Currently known manganese oxides that can exist stably at room temperature are the following: MnO_2_, Mn_5_O_8,_ Mn_2_O_3_, Mn_3_O_4_ and MnO(Pu et al., 2020). Kapteijn et al. (Kapteijn et al., 1994) reported that MnO_2_ has the highest low-temperature activity per unit surface area, and Mn2O3 has the highest N_2_ selectivity. For the single oxide MnO_x_, the crystallinity and valence state have a great influence on the SCR performance. Typically, crystallinity depends on the preparation and calcination temperature. Tang et al. (Tang et al., 2007) prepared manganese oxide catalysts by rheological phase reaction (RP), low temperature solid phase reaction (SP), coprecipitation (CP) and citric acid (CA). The results show that the Mn_2_O_3_ produced by the CA has high crystallinity, only a part of MnOx produced by the RP, and the MnO_x_ produced by the SP and the CP is transformed into an amorphous state. The lower the crystallinity of MnO_x_, the higher the low temperature SCR performance. Kang et al. (Kang et al., 2006; Kang et al., 2007) prepared manganese oxide catalysts by precipitation and investigated the effects of precipitants and calcination temperature on catalyst performance. It has been reported that the catalyst prepared by the sodium carbonate precipitation (MnO_x_-SC) has a higher specific surface area than other technologies, which may be one of the reasons for its excellent performance. In addition, the partial decomposition and amorphous structure of the MnO_x_-SC catalyst may also account for its relatively high activity. Tang et al. (Tang et al., 2010) prepared MnO_x_ with different crystal forms and valence states by redox, and studied their SCR performance. The results showed that the NOx conversion rate and N_2_O generation rate per unit surface area on β-MnO_2_ were higher than those on α-Mn_2_O_3_. The corresponding value on β-MnO_2_ is much higher, the reason why β-MnO_2_ exhibits higher N_2_O selectivity is that its lower Mn-O bond energy promotes the activation of NH_3_. Although manganese-based materials exhibit high catalytic performance at low temperatures, they exhibit many disadvantages in practical applications, such as sensitivity to SO_2_ and low N_2_ selectivity (Cai et al., 2014). Therefore, loading MnOx on stable supports or doping MnOx with other elements is a general strategy to prepare high-performance and high-selectivity SCR catalysts. Peña et al. (Peña et al., 2004) pointed out that using solution impregnation to support various transition metal oxides on TiO2, manganese-based catalysts are the best candidates for low-temperature SCR reaction, and their activities decrease in the following order: Mn > Cu ≥ Cr » Co. > Fe » V » Ni. In addition, the activated carbon-supported manganese-based monolith catalyst (MnO_x_/AC) (Huang et al., 2008) prepared by impregnation, and the mesoporous silica-supported iron-manganese composite oxide (Mn-Fe/MPS) catalyst both showed excellent SCR performance. For Mn-based composite metal oxides, Mn-Fe composite oxide catalysts have been reported to exhibit good SCR activity, high N_2_ selectivity, and ideal low-temperature water-sulfur poisoning resistance (Lin et al., 2006). Wu et al. (Wu et al., 2008) found that the addition of Fe promoter can increase the number and strength of Brönsted and Lewis acid centers on the surface of Mn-based catalysts, and promote the adsorption of NH_3_ to further form some active intermediates, thereby improving the low-temperature performance of NH_3_-SCR. Furthermore, MnO_x_-CeO_2_ binary oxide catalysts have attracted much attention due to their high NOx removal efficiency at temperatures below 250 °C. This is based on the oxygen storage capacity and redox properties of CeO_2_, that is, the redox properties between Ce^4+^ and Ce^3+^ to store or release oxygen (Yan et al., 2020). Liu et al. (Pu et al., 2020) synthesized a series of Mn-Ti oxide catalysts by sol-gel, and investigated the effect of different synthesis conditions on catalyst performance. The results show that calcination temperature and metal source have important effects on catalyst performance and SCR activity. The lower calcination temperature is beneficial to improve the low temperature SCR activity. With manganese nitrate or manganese acetate as manganese source and tetrabutyl titanate as titanium source, higher SCR activity can be obtained. Its excellent low-temperature NH_3_-SCR activity can be attributed to good texture properties, comparable ratios of Mn^3+^/Mn^4+^ in amorphous Mn oxides, good low-temperature reducibility, and abundant surface Brönsted acid sites. Gao et al. (Gao et al., 2020) investigated the low-temperature catalytic reduction of NO on novel Mn-Ni spinel nanosheets by the urea hydrolysis (UH) and the urea hydrolysis hydrothermal synthesis (UHHS), and combined with the coprecipitation hydrothermal synthesis (CPHS) Compared with coprecipitation (CP). As shown in Figures 4A–C, the results determined the optimal Mn/Ni (2:1) and urea/(Mn + Ni) (3:1) molar ratios, the optimal hydrothermal temperature (130°C, 24 h) and Calcination temperature (450°C, 6 h). Compared with the Mn (2)Ni(1)O_x_-UH catalyst, the Mn (2)Ni(1)Ox-UHHS preparation yielded pure spinel-structured NiMn_2_O_4_ nanosheets, both of which exhibited highly efficient SCR activity (100–250°C, >98% NO_x_ conversion) and N_2_ selectivity (>95% at <150°C, >85% at <250 °C). NiMn_2_O_4_-UHHS nanosheets also exhibited excellent resistance to H_2_O and SO_2_ in the low temperature range (85–90% NO_x_ conversion at 150–300°C, 10 vol% H_2_O and 150 ppm SO_2_). Zhao et al. (Zhao et al., 2020) prepared tourmaline-modified CeMnFeOx low-temperature catalysts by hydrothermal. The results show that when the addition amount of tourmaline is 2%, the catalytic performance of the composite catalyst is the best, and its NOx conversion rate is 100% at 170–230°C.

**FIGURE 5:**
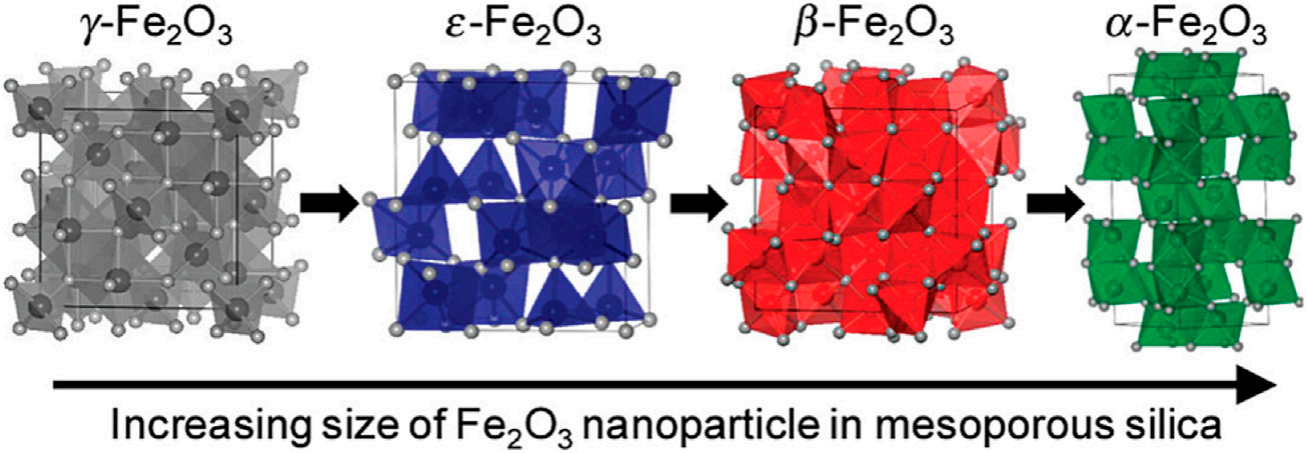
Schematic diagram of the structure of four different crystal forms of Fe_2_O_3_, (the colored ball in the center of the polyhedron represents the Fe atom, and the white atom on the edge represents the oxygen atom (Sakurai et al., 2009).

Iron-based catalysts, as environmentally friendly and low-cost catalysts, exhibit very active and stable catalytic performance during the SCR reaction due to their good redox capacity (Yan et al., 2015; Zhang et al., 2017). For example, based on isotopic labeling, iron-based catalysts exhibit stable NH_3_-SCR performance in the presence of water vapor (Sun et al., 2001). However, the NO_x_ removal efficiency of pure α-Fe_2_O_3_ is not satisfactory, especially the low temperature performance. Therefore, most of the research work has focused on tuning the crystal phase/plane and nanostructure of α-Fe_2_O_3_, while its thermal stability and acidity/redox performance can also be improved by modification or doping. Crystallographic phase/plane and morphology control of Fe_2_O_3_ nanomaterials is an important research topic in the fields of materials science and catalysis. In Fe_2_O_3_, according to the arrangement of Fe^3+^ and O^2-^, it can be divided into four crystal types: α, β, γ, ε, and the crystal structure is shown in Figure 5. Among the four different crystal structures, two types of Fe_2_O_3_ are commonly used in catalytic reactions, namely hematite (α-Fe_2_O_3_) and maghemite (γ- Fe_2_O_3_), and their crystal structures and morphology have a significant impact on SCR performance. In contrast, the γ- Fe_2_O_3_ catalyst exhibited better SCR activity than α-Fe_2_O_3_ at 150–300°C. For example, Shen et al. (Mou et al., 2012) prepared rod-like γ-Fe_2_O_3_ with preferentially exposed (110) and (001) crystal planes by aqueous precipitation, and the results showed excellent NH3-SCR performance. In addition, Dong et al. (Yu et al., 2021) used γ-Fe_2_O_3_ as a model catalyst to study the effect of reaction temperature on catalytic activity in the presence of SO_2_. The results showed that the introduction of SO2 had no negative effect on the activity at 300°C, and the activity gradually increased with time. At low temperature (225–275°C), the SCR activity first decreased and then increased slowly. This is mainly due to the fact that at low temperature, the sulfate formed on the catalyst surface inhibits the adsorption of NO_x_, cutting off the L-H reaction pathway, resulting in the initial drop of NOx. However, with the continuous progress of the reaction, the deposited ammonium bisulfate (ABS) can be continuously consumed by NO + O_2_, indicating that the formation and consumption of ABS have reached a dynamic equilibrium. In addition, the formation of ferric sulfate species led to the enhancement of surface acidity, which facilitated the E-R reaction pathway, which further contributed to the enhanced activity. Liu et al. (Liu et al., 2017) prepared nanoporous α-Fe_2_O_3_ with different surface areas by thermal treatment of α-FeOOH. They found that increasing the heat treatment temperature could improve the crystallization of α-Fe_2_O_3_ and reduce surface oxygen defects, resulting in lower activity.

**FIGURE 6:**
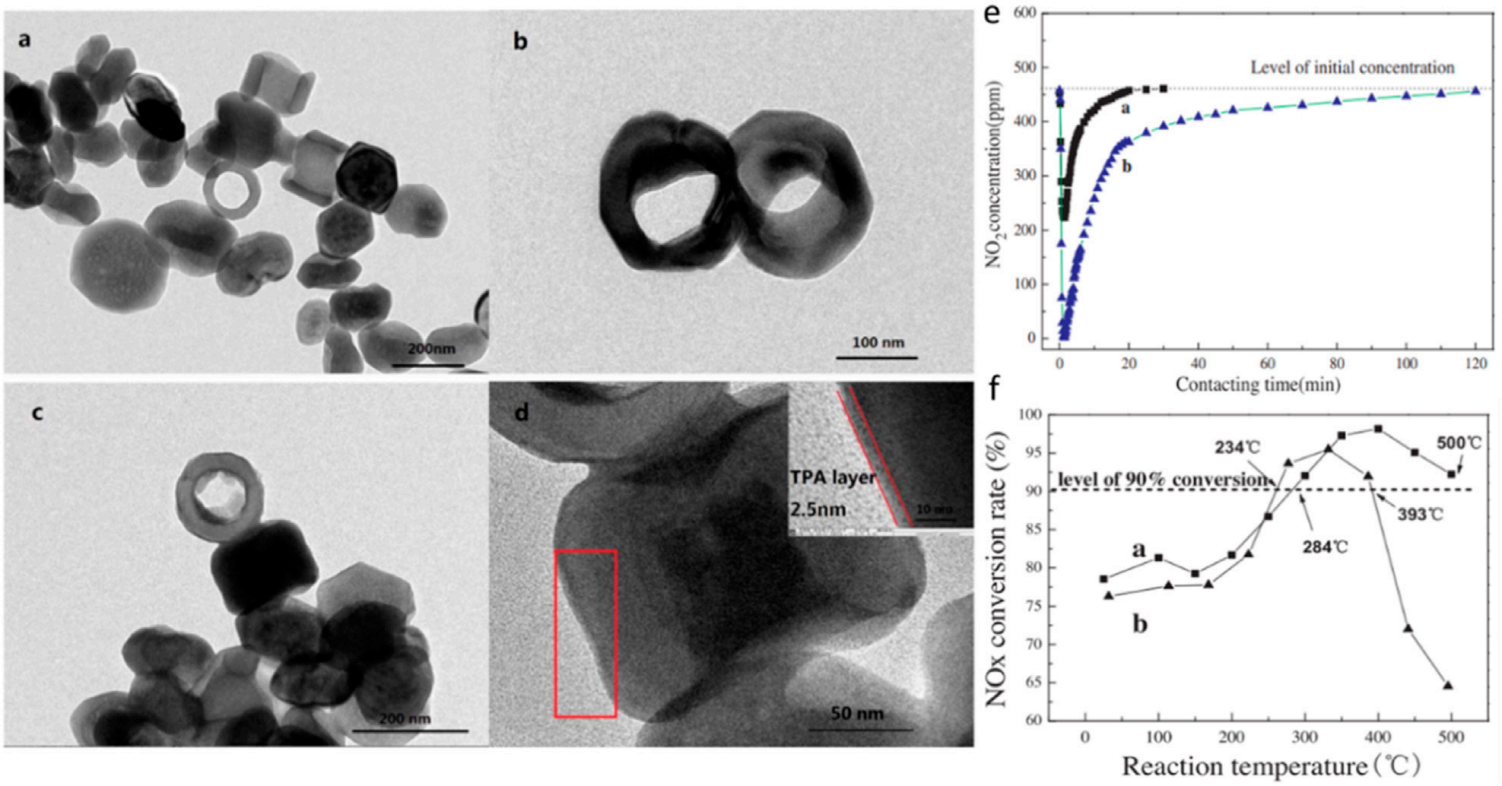
Microwave-assisted loading of phosphotungstic heteropolyacids on Fe_2_O_3_ surfaces: **(A–D)** Microstructure: **(E)** NO_2_ adsorption effect; **(F)** NO_x_ removal effect (Ren et al., 2017b).

For supported Fe_2_O_3_ catalysts, Fe_2_O_3_ can be used as the active component or itself as the support. When Fe_2_O_3_ is used as the active component, the interaction between Fe_2_O_3_ and the support can be adjusted by adjusting the crystal plane of the support, thereby adjusting the SCR activity of the catalyst (Han et al., 2016). For example, Zhang et al. (Xin et al., 2018a) prepared TiO_2_-NS (nanosheets) and TiO_2_-NSP (nanoshafts) supported monolayer Fe_2_O_3_ catalysts by the isometric impregnation, since Fe_2_O_3_/TiO_2_-NS has more acid sites, oxygen defects and reactive oxygen species, thus exhibiting better low temperature activity. In addition, they also prepared CeO_2_-NR (nanorod) and CeO_2_-NPH (nanopolyhedron) supported Fe_2_O_3_ catalysts by hydrothermal synthesis. Among them, CeO_2_-NR mainly exposes (110) and (100) planes, while CeO_2_-NPH exposes 111) planes, and it was found that Fe_2_O_3_/CeO_2_-NR has higher activity than Fe_2_O_3_/CeO_2_-NPH(Liu et al., 2016). When Fe_2_O_3_ is used as a carrier, it has been mentioned that Fe_2_O_3_ itself has good Fe^3+^/Fe^2+^ switching ability, but its surface acidity is relatively poor. Due to redox properties and acidity are essential for SCR catalysts. Therefore, the support of acidic oxides on Fe_2_O_3_ is also a topic of concern to many researchers. For example, Xin et al. used the co-precipitation to doped Fe_2_O_3_ with acidic Mo (Xin et al., 2018a) and W (Xin et al., 2018b) atoms to prepare SCR catalysts, which showed better SCR performance than pure Fe_2_O_3_. The catalyst prepared by Liu et al. (Liu F. et al., 2018) supported acidic WO_3_ on Fe_2_O_3_ by impregnation has a wide temperature window and excellent water and sulfur resistance. The introduction of WO_3_ can not only maintain the proper redox performance of the catalyst, but also enhance the acidity (Lewis) of the catalyst and promote the adsorption and activation of NH3. At the same time, WO_3_ can also inhibit the crystallization of Fe_2_O_3_ and increase the specific surface area of ​​the catalyst. In addition, He et al. (Ren et al., 2017a) used the impregnation to disperse WO_3_ on the surface of α- Fe_2_O_3_, and studied the interaction between WO_3_ and α- Fe_2_O_3_ in detail, and found that WO_3_ was dispersed on the surface of Fe_2_O_3_ in the form of unsaturated coordination. The unsaturated coordination of WO_3_ enhances the oxidative capacity of surface Fe through the electronic effect between Fe-O-W, thus facilitating the oxidative dehydrogenation of NH_3_. At the same time, the excessive oxidation of bulk Fe at high temperature to form harmful by-product N_2_O is also suppressed. Similar to WO_3_/MoO_3_, heteropolyacids have also been used in SCR reactions due to their good thermal stability and high acidity. Ren et al. (Ren et al., 2017b) used a microwave-assisted technology to load phosphotungstic heteropolyacids on the surface of Fe_2_O_3_, as shown in Figure 6. The results show that the modified catalyst exhibits >90% NO_x_ conversion and good water and sulfur resistance at 250–500°C due to the interaction between Fe^3+^ and heteropolyacids.

(3) Molecular sieve catalyst

**FIGURE 7:**
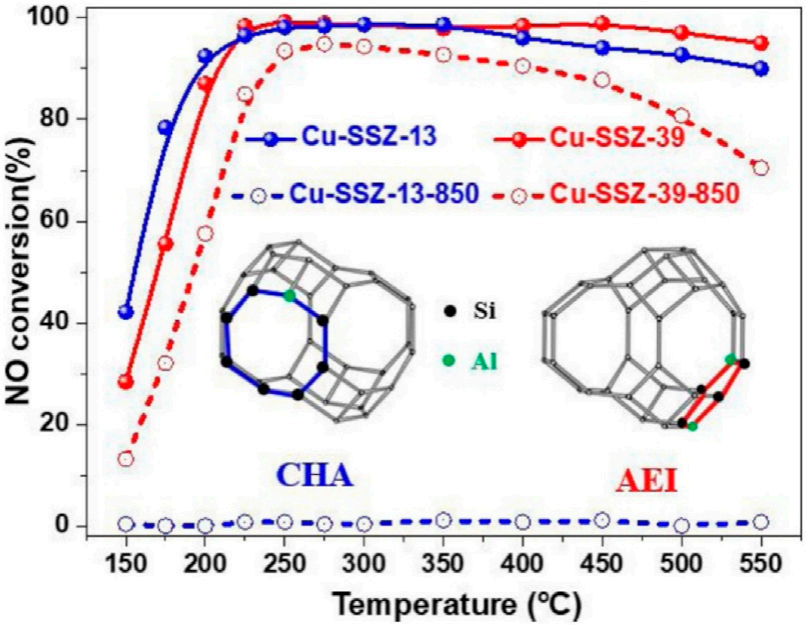
Comparison of NH_3_-SCR performance of Cu-SSZ-13 and Cu-SSZ-39 after high temperature hydrothermal treatment at 850°C (Shan et al., 2020).

Molecular sieves have special pore structure and surface properties, and are widely used in the field of catalysis. They are usually used as catalyst supports in SCR denitration. The commonly used molecular sieves are mainly zeolites, including ZSM, Y-zeolite, MOR and MIF. The molecular sieve catalysts have high denitrification and have attracted attention in the research of catalytic NOx reduction (Xu et al., 2019). To meet the increasingly stringent emission regulations, many researchers have been looking for new molecular sieve catalysts with higher hydrothermal stability and excellent catalytic performance. Hong et al. (Ahn et al., 2017) synthesized high-silicon (Si/Al>8) LTA molecular sieves with tetramethylammonium ions using benzylimidazolyl OSDA as a structure-directing agent in a fluoride medium. Importantly, it still exhibits excellent NOx removal efficiency even after hydrothermal aging at 900°C after exchanging Cu^2+^ ions. In addition, they also explored the essential reasons for the high hydrothermal stability of high-silicon LTA using a series of characterizations. It was found that in the high-silicon Cu-LTA zeolite, Cu^2+^ ions were not only neutral in catalytic activity, but also located at the center of a single six-membered ring (S6RS), acting as a dealumination inhibitor of the zeolite (Ryu et al., 2019). As another new type of molecular sieve, Cu-SSZ-39 (AEI configuration) is very similar in structure to Cu-SSZ-13 (CHA type), and also exhibits excellent hydrothermal stability. The only difference between the AEI and CHA structures is the way the double 6-ring (D6R) is attached. Adjacent D6Rs have mirror symmetry in the AEI but are aligned in parallel in the CHA, thus resulting in different AEI and CHA cavities (Shan et al., 2020). In recent years, Cu-SSZ-39 has received extensive attention in the NH3-SCR reaction. Moliner and Corma et al. found that Cu-SSZ-39 exhibited high SCR activity and hydrothermal stability. He et al. (Shan et al., 2020) compared the NH_3_-SCR performance of low Si/Al Cu-SSZ-13 and Cu-SSZ-39 after high temperature hydrothermal treatment at 850°C, as shown in Figure 7. The results show that Cu-SSZ-39 exhibits more excellent resistance to high temperature hydrothermal. In addition, their research group also explored the effect of the presence of water vapor on the sulfation of Cu-SSZ-39 catalyst on its NH_3_-SCR catalytic performance at different temperatures (200°C, 400°C, 600 °C). The results show that sulfation at 200 and 400°C leads to a significant decrease in SCR performance, while at 600°C, the sulfation phenomenon is not severe. This is mainly due to the H_2_SO_4_ covering the active centers at 200 and 400°C, and the formation of stable CuSO_4_ species is also the main reason for the deactivation of the sulfated catalysts at 200°C, 400°C, and 600°C. The activity of the sulfated catalyst could be partially recovered after regeneration at 600°C, but was still lower than that of the fresh catalyst due to the formation of stable copper sulfate species (Du et al., 2020). The main disadvantages of the current molecular sieve catalysts include SO_2_ poisoning, carbon deposition, hydrothermal aging and so on. If the large-scale industrial application of molecular sieve catalysts is to be realized, in-depth research and exploration are still required.

(4) Carbon-based catalyst

Activated carbon fiber ACF has a well-developed microporous structure and excellent adsorption performance, which is suitable as a catalyst carrier, and the functional groups contained on its surface are conducive to the progress of the catalytic reaction. Therefore, using ACF as a catalyst carrier to develop SCR catalysts with lower catalytic temperature and high performance has become a topic now. The study found that Fe_2_O_3_/AC (Yang et al., 2016) and Fe_2_O_3_/ACF(Wang and Gui, 2013) catalysts have good SCR denitrification activity below 200°C, CeO_2_/ACF(Zeng et al., 2012)at 120–270°C. The denitrification efficiency of CeO_2_-Fe_2_O_3_/ACF(Wang and Gui, 2013) was maintained above 96% in the range of 80–320°C, and the V_2_O_5_/AC catalyst could simultaneously desulfurize and denitrify at 200°C, and showed good catalytic performance. However, carbon-based catalysts will suffer losses during the reaction process, are flammable at 250°C, and contain metal impurities that will affect the catalytic activity, limiting the application potential to a certain extent.

In the development process of SCR denitration reaction, it is a process of developing from high temperature reaction SCR catalyst to low temperature reaction. The industry has begun to improve the V_2_O_5_/TiO_2_ system catalyst. Traditional industrial catalysts are often placed before dust collectors and desulfurization devices, resulting in Sulfurization and deactivation of the catalyst and the blockage of the catalyst by flue gas impurities affect the service life of the catalyst and cause unnecessary losses in terms of economy and environmental protection (Zhe et al., 2007). Therefore, the production of a denitrification system for dust collectors and desulfurization devices is an effective way to solve this problem. The temperature at the end of the pipeline is often lower than 200°C, and it is necessary to re-select and adjust the active components and carriers of the catalyst in the existing system.

## Deactivation mechanism and regeneration of SCR denitration catalysts

In the SCR denitration system, the catalyst, as the core component, is usually placed before the air preheater, that is, the arrangement of high temperature and high dust. When the catalyst operates under such conditions, the catalyst will be deactivated due to poisoning, clogging, sintering and other reasons in the environment of high temperature and harmful substances such as fly ash and impurities for a long time. If the catalyst is arranged in a low-temperature and low-dust arrangement, that is, after the desulfurization and dust removal device, the catalyst can avoid problems such as sintering caused by high-temperature flue gas and clogging of catalyst pores by fly ash. Therefore, it is particularly important for the regeneration of deactivated catalysts and the development of medium and low temperature (<300°C) SCR denitration catalysts. Wide-temperature denitration catalysts are easily affected by H_2_O and SO_2_ in applications, resulting in reduced activity and shortened service life. The modification of wide temperature catalysts for SO2 poisoning resistance and ammonium bisulfate (ABS) deposition ability has been the focus of research in this field.

### Deactivation mechanism of SCR denitration catalysts

For SCR denitration catalysts in coal-fired power plants, the reasons for catalyst deactivation are different due to different operating conditions. Studies have shown that catalyst deactivation mainly has two aspects, one is the deactivation caused by physical factors, and the other is the deactivation caused by chemical substances. The physical factors of catalyst deactivation mainly include:

(1) Wear of the catalyst

The wear of the catalyst is mainly due to the fact that the catalyst is in the scouring state of fly ash and impurities for a long time. When the flow rate of the flue gas is too fast and the impact angle of the fly ash is not appropriate, the wear degree of the catalyst will be aggravated. And the properties of the catalyst will also affect the degree of wear of the catalyst (Liu et al., 2020). Therefore, in order to reduce the wear degree of the catalyst, the flow field of the denitration reaction can be adjusted and optimized or the linear velocity of the flue gas flow can be changed.

(2) Catalyst pores are blocked

**FIGURE 8:**
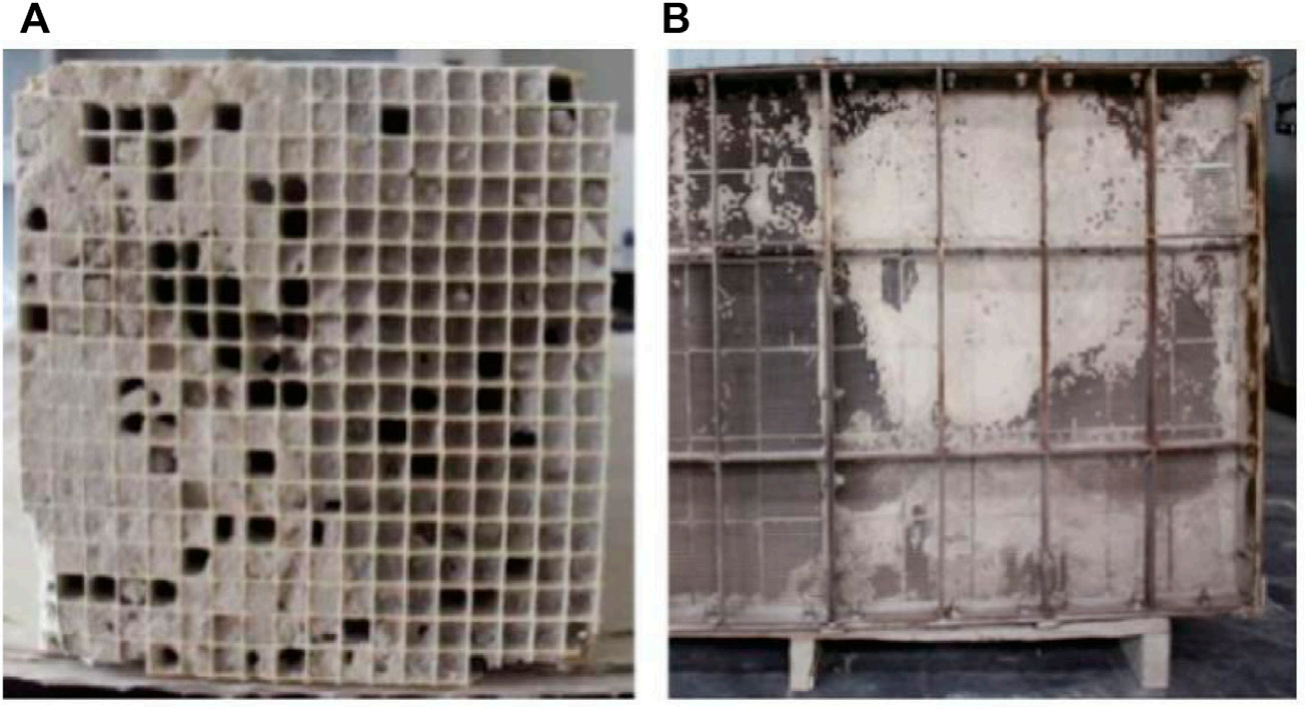
SCR catalyst plugged **(A)** and deactivated **(B)** (Li et al., 2016).

The blockage of the catalyst pores is mainly due to the fact that small particles of fly ash and ammonium salts (NH_4_HSO_4_ and (NH_4_)_2_SO_4_) gradually accumulate in the catalyst pores during the long-term operation of the catalyst, and the particles gradually cover the surface of the pores from small to large, resulting in smoke The non-circulation of gas components (as shown in Figure 8) will correspondingly reduce the denitration activity of the catalyst (Liu et al., 2020).

(3) Catalyst sintering

The sintering of the catalyst is mainly due to the change of the crystal phase of the active components on the surface of the catalyst when the catalyst is kept at an excessively high temperature for a long time, thus affecting the denitration activity of the catalyst. The crystal phase of the catalyst carrier TiO_2_ is mainly anatase type, but too high temperature will cause the anatase type TiO_2_ to start to transform into rutile phase. The number of catalyst micropores will be reduced accordingly, the specific surface area will also be reduced accordingly, and the overall denitration activity of the catalyst will be significantly reduced (Lu et al., 2020). The chemical factors of catalyst deactivation mainly include:

(1) Alkali metal poisoning

**FIGURE 9:**
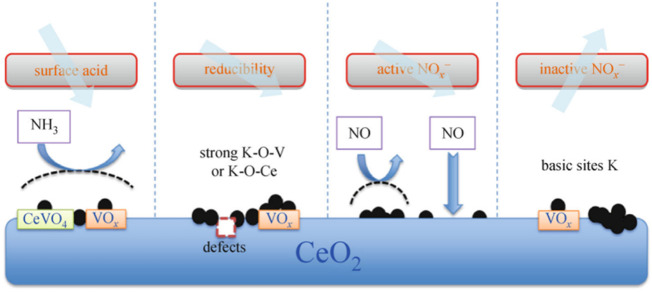
K_2_O poisoning mechanism on CeO_2_ surface (Li et al., 2016).

Alkali metal poisoning refers to the binding of alkali metals to the active sites of catalysts. Alkali metal species can cover and occupy the original active sites on the catalyst surface, resulting in catalyst deactivation (Chang et al., 2018; Chen et al., 2018). Yue et al. (Peng et al., 2015) studied the deactivation mechanism and regeneration of alkali metal and arsenic poisoned catalysts, and analyzed V_2_O_5_-WO_3_/TiO_2_ catalysts by experiments and density functional theory. The results showed that alkali metal poisoning mainly reduced the Brønsted acid site of the catalyst. The amount and reducibility of active V^5+^. Chen et al. (Chen et al., 2018) studied the deactivation effect of potassium on SCR denitration catalysts, and the study showed that the presence of potassium had an inhibitory effect on the SCR activity of the catalyst, as shown in Figure 9. Potassium mainly inhibits the oxidation of NO to NO_2_ and promotes the conversion of NH_3_ to NO_x_ and N_2_O, which is the main reason for the significant decrease of both the low-temperature SCR activity and N_2_ selectivity of SCR denitration catalysts.

(2) Acid poisoning

The acidic substances contained in the catalyst mainly include elements such as S, Cl and P and their derivatives. During the operation of the SCR denitration device, if the denitration reactor is placed before the desulfurization device, the flue gas is not desulfurized, which will lead to a large amount of SO_2_ in the flue gas. SO_2_ is oxidized to the more harmful SO_3_ under the influence of catalyst substrates and other oxidizing substances. The sulfate formed by SO_3_ can lead to the blockage of catalyst pores and the sulfation of active sites, which seriously affects the SCR denitration reaction. SO_2_ and water vapor are the main causes of deactivation of low temperature SCR catalysts. Sheng et al. (Sheng et al., 2012) prepared a SCR denitration catalyst by co-precipitation. By simulating the poisoning components in the flue gas, the catalyst was poisoned and its deactivation mechanism was studied. Studies have shown that the presence of SO_2_ can react with the active components in the catalyst to form stable sulfate and ammonium salts, etc., resulting in a decrease in catalyst activity and difficult recovery. During the selective reaction of NH_3_ with NO, the presence of H_2_O inhibits the process. The NH_3_ retained in the flue gas also reacts with SO_3_ to form ammonium sulfate salt, resulting in a significant decrease in the denitration activity of the catalyst (Qi et al., 2019).

Through the summary of the above-mentioned poisoning mechanism of SCR denitration catalyst, it is found that in order to improve the catalyst’s anti-sulfur and water-resistance performance, it is necessary to overcome the two problems of the deposition of ammonium sulfate and the sulfation of active metal atoms. In the actual operating conditions of the power plant, the actual flue gas temperature may be lower than the set value, and the reduction of the flue gas temperature is conducive to alleviating the sulfation of active metal atoms, but is not conducive to the decomposition of ammonium sulfate. Therefore, the development of medium and low temperature catalysts with good sulfur resistance and water resistance is a common problem facing environmental protection at present.

### Regeneration of SCR denitration catalysts

Typically, denitration catalysts in coal-fired power plants will deactivate after 24,000 h of operation. Catalyst is the core of SCR flue gas denitration technology, and its cost accounts for more than 40% of the investment cost of the entire system. Generally, catalysts used in industrial applications need to be replaced every 2–3 years. A large number of waste catalysts have caused huge pressure on environmental protection, and the regeneration price is relatively low. Therefore, the industrialization of regeneration technology has far-reaching significance. There are many reasons for the deactivation of catalysts in industrial applications, mainly including As, alkali metal (Na, K) poisoning, sulfur poisoning, fouling and clogging, sintering and volatilization of active components, and mechanical wear. The industrial process route of catalyst regeneration is: catalyst deactivation cause diagnosis, cleaning, loosening, compound pore, strengthening, activation, heat treatment. According to the “Technical Policy for the Prevention and Control of Nitrogen Oxides in Thermal Power Plants”, the preparation of high-performance catalyst raw materials, new catalysts and regeneration of deactivated catalysts are encouraged. The regeneration process is mainly to remove the blocking substances accumulated inside the catalyst by cleaning the deactivated components of the catalyst, so that the SCR denitration reaction can be carried out normally. For different catalyst deactivation reasons, it is the key to the regeneration process to take corresponding and effective regeneration to restore the catalyst activity. As shown in Table 1, common SCR catalyst regeneration.

**TABLE 1:** Advantages and disadvantages of technologies for SCR catalyst regeneration.

Technology	Advantages	Disadvantages
Washing and regeneration	Simple and efficient	Unable to completely restore the catalyst activity of alkali poisoning
Pickling regeneration	Good regeneration effect on alkali metal poisoning	Leads to the loss of active components on the surface of the catalyst
		Introduces surface sulfate, which causes corrosion to the equipment generator
		Produces a large amount of waste liquid
Alkaline washing regeneration	High regeneration efficiency	Causing certain damage to the catalyst, resulting in a large amount of waste liquid
	Less corrosion to the engine	
SO2 acidification thermal regeneration	Improve catalyst surface acid sites	The regeneration efficiency is not high

Among them, water washing is the simplest and most widely used regeneration. The basic principle is to rinse the catalyst with deionized water to remove some toxic substances such as ammonium sulfate on its surface, and at the same time, it can improve the surface microscopic morphology of the catalyst. At present, there are many studies on the regeneration of sulfur poisoning by water washing, and different catalysts have good regeneration effects. Generally speaking, for pore blockage caused by calcium sulfate, ammonium salt, etc. and chemical poisoning caused by alkali metals and arsenic substances, the purpose of regeneration cannot be achieved by simple water washing treatment, and a specific regeneration solution needs to be prepared for cleaning. For example, surfactant solutions such as inorganic acids, emulsions, permeates, and complexing liquids. Studies have shown that inorganic acids such as H_2_SO_4_, HNO_3_ and HCl can effectively wash away the alkali metal species (Na_2_O and K_2_O) on the catalyst surface (Li et al., 2017). If the catalyst P is poisoned, it can be immersed in alkaline solution and cleaned by ultrasonic wave, which can restore the activity of the catalyst to 80–90%. For catalyst as poisoning, NaOH, Na_2_CO_3_ and NaHCO_3_ solutions can be used for alkaline washing, and then used for HNO3 acidification. While As is removed during the regeneration process, the active components of the catalyst are effectively retained (Yu et al., 2013).

**FIGURE 10:**
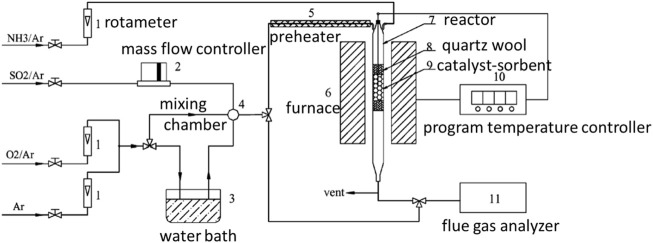
Schematic diagram of the desulfurization regeneration experimental device (Xie et al., 2003).

In recent years, researchers have developed some new regeneration for catalyst regeneration, such as thermal regeneration, thermal reduction regeneration, and SO_2_ acidification thermal regeneration (Zheng et al., 2004). The thermal regeneration refers to placing the deactivated catalyst in an atmosphere of inert gas, and heat-treating the catalyst at a certain heating rate. The complete decomposition temperature of ammonium sulfate is above 400°C, so the temperature of thermal regeneration is usually above 400°C. The ammonium salt formed on the surface of the catalyst will gradually decompose, so that the denitration activity of the catalyst can be restored. The thermal reduction regeneration refers to that on the basis of heat treatment, the deactivated catalyst is placed in a mixed gas atmosphere of inert gas and reducing gas (H_2_ and NH_3_), and the high-valent sulfur on the surface of the catalyst is reduced under high temperature conditions. Restore the denitration performance of the catalyst. After the V_2_O_5_/AC catalyst was regenerated at 300 °C in an atmosphere of 5%NH_3_-95%Ar, the performance of the catalyst was recovered well. The analysis considered that the content of NH^4+^ and NH^2-^ on the catalyst surface increased, forming new active sites (Cao et al., 2019; Li et al., 2020). However, some studies have also found that the catalyst activity decreases after thermal reduction regeneration with NHs, which may be because NH_3_ and SO_2_ adsorbed on the surface of the catalyst generate ammonium sulfate, which causes the blockage of the pores again. Xie et al. (Xie et al., 2003) studied the thermal reduction regeneration of CuO/A1_2_O_3_ catalyst with 5% NH_3_-95% Ar. Using the thermal regeneration equipment shown in Figure 10, the CuO component can be effectively regenerated at 400°C, while the regeneration effect of the A1_2_O_3_ component is not. ideal. The SO_2_ acidification thermal regeneration mainly places the catalyst in the SO_2_ atmosphere to restore the denitration activity of the catalyst, which is mainly aimed at the alkali metal poisoning of the catalyst.

## Summary and outlook

Among many NO_x_ control technologies, SCR technology is currently the most effective and internationally recognized NOx elimination technology. Among them, the catalyst is the key in this technology. Therefore, the development of high-performance SCR catalysts and the in-depth study of their active sites are of great theoretical and social significance for guiding the design of new SCR catalysts and their introduction to the market. Considering the operating cost of coal-fired power plants and the importance of environmental protection, regeneration of deactivated catalysts is necessary. By consulting and reading a large number of literatures, this paper summarizes the current research hotspots of SCR catalysts and puts forward the problems and challenges faced by the current SCR catalysts.

Some interesting results have been obtained on the influence of catalyst SCR reactivity and the tentative design of mesoporous structure sulfur-tolerant catalysts. However, for the practical application of catalysts, the development of highly stable catalysts that meet the harsh industrial flue gas environment still faces great challenges, and there are some key scientific issues that need to be solved urgently. The following aspects still need to be further improved and developed in the follow-up work:

(a) Development of high-performance wide-temperature denitration catalysts. Coal quality in various countries is complex. Alkali coal, high arsenic coal, high mercury, high sulfur and high ash coal will directly cause the flue gas of coal-fired power plants to contain a variety of components that can easily lead to catalyst poisoning. The policy encourages the adoption of the “biomass utilization + incineration” disposal model, which uses waste incineration power plants, coal-fired power plants, cement kilns and other synergistic sludge and biomass fuels as a supplement to its disposal. The composition of flue gas produced by mixing sludge and biomass fuel is relatively complex, except for nitrogen oxides and sulfur oxides, as well as chlorides and heavy metals. Therefore, it is necessary to continuously consider the composition of catalytic materials and the design of surface microstructures. The innovative materials solve the problems of catalyst low-temperature denitration activity, hydrophobicity and ammonium salt deposition poisoning resistance when the unit is running at low load. Adapt to alkali and heavy metal components in flue gas and slow down catalyst failure.
(b) Carry out research on catalytic processes under complex flue gas conditions. Comprehensively carry out catalyst performance evaluation tests under actual flue gas conditions (low temperature, high dust, high sulfur, etc.), and establish the relationship between reaction temperature, space velocity, water vapor, SO_2_ and other parameters and catalyst activity and stability. The popularization and application of denitration catalysts provide reliable basic data. The honeycomb catalyst extrusion molding process has matured. However, this process has strict requirements on the amount of active components and production process parameters, and it is difficult to form a honeycomb catalyst with a high pore number. The coating process can significantly save raw materials and reduce the cost of catalysts, and has the advantages of simple process operation, high strength and easy regeneration. This is suitable for the manufacture of honeycomb catalysts with high pore number and high content of active components, and can meet the denitration needs of flue gas in different fields.
(c) Develop a selection and design for wide-temperature denitration catalysts. According to the flue gas conditions of coal-fired power plants, rational design of denitration catalyst is the key to the normal operation of denitration system. Screen catalyst formulations based on big data and consider the impact of specific flue gas conditions. Calculate the technical parameters such as catalyst dosage, active component content, ammonia consumption in system operation, ammonia injection temperature, catalyst arrangement and catalyst life, and obtain catalyst design and selection parameters that meet engineering conditions and can be used for flue gas denitrification. On the premise of meeting the denitration performance requirements, the loss and accident rate should be minimized, the system operation efficiency should be improved, and the operation cost of the denitration system should be reduced.
(d) Exploring *in-situ*/off-line regeneration for wide-temperature denitration catalysts. Through experiments and theoretical calculations, the formation and accumulation conditions (temperature, space velocity, NO/NO_2_, water vapor, etc.) of ABS in the catalyst and reaction system under low temperature conditions were obtained, and the safety of wide-temperature SCR catalysts in low temperature operation was comprehensively evaluated. At the same time, *in-situ* analysis and technologies based on the characteristics of different materials and poisoning substances are developed to prolong the service life of catalysts.
(e) Implement full-life management of denitration catalysts. The investment cost of the wide temperature denitration catalyst is high, and the catalyst has a limited service life. After the expiration date, a new catalyst needs to be installed or replaced, increasing the investment cost. Therefore, the service life of the catalyst should be extended by strengthening the catalyst management and standardizing the whole life management process of installation, operation, testing and maintenance. With the increase of operating time, the deactivation of the catalyst is inevitable, and the life-cycle management of the denitration catalyst is of great significance to prolong the service life of the catalyst. Waste catalysts are classified as hazardous solid wastes, which are difficult to deal with. Strengthening the research and development of waste catalyst treatment technology, including catalyst secondary regeneration technology, extraction of trace elements from waste catalyst, and recovery of effective components are the future development trends.

## References

[B1] Ahn N. H. Ryu T. Kang Y. Kim H. Shin J. Nam I.-S. (2017). The origin of an unexpected increase in NH3–SCR activity of aged Cu-LTA catalysts. ACS Catal. 7, 6781–6785. 10.1021/acscatal.7b02852

[B2] Albert K. B. Fan C. Pang L. Chen Z. Ming S. Albert T. (2019). The influence of chemical poisoning, hydrothermal aging and their co-effects on Cu-SAPO-34 catalyst for NOx reduction by NH3-SCR. Appl. Surf. Sci. 479, 1200–1211. 10.1016/j.apsusc.2019.02.120

[B3] Andana T. Rappe K. G. Gao F. Szanyi J. Pereira-Hernandez X. Wang Y. (2021). Recent advances in hybrid metal oxide–zeolite catalysts for low-temperature selective catalytic reduction of NOx by ammonia. Appl. Catal. B Environ. 291, 120054. 10.1016/j.apcatb.2021.120054

[B4] Cai S. Zhang D. Shi L. Xu J. Zhang L. Huang L. (2014). Porous Ni–Mn oxide nanosheets *in situ* formed on nickel foam as 3D hierarchical monolith de-NO x catalysts. Nanoscale 6, 7346–7353. 10.1039/C4NR00475B 24861850

[B5] Cai T. Zhao D. Sun Y. Ni S. Li W. Guan D. (2021). Evaluation of NO emissions characteristics in a CO2-Free micro-power system by implementing a perforated plate. Renew. Sustain. Energy Rev. 145, 111150. 10.1016/j.rser.2021.111150

[B6] Cao Y. Han F. Wang M. Han L. Zhang C. Wang J. (2019). Regeneration of the waste selective catalytic reduction denitrification catalyst by nitric acid washing. ACS omega 4, 16629–16637. 10.1021/acsomega.9b02288 31616845 PMC6788065

[B7] Casanova M. Rocchini E. Trovarelli A. Schermanz K. Begsteiger I. (2006). High-temperature stability of V2O5/TiO2-WO3-SiO2 SCR catalysts modified with rare-earths. J. alloys Compd. 408, 1108–1112. 10.1016/j.jallcom.2004.12.128

[B8] Cavataio G. Jen H.-W. Girard J. W. Dobson D. Warner J. R. Lambert C. K. (2009). Impact and prevention of ultra-low contamination of platinum group metals on SCR catalysts due to DOC design. SAE Int. J. Fuels Lubr. 2, 204–216. 10.4271/2009-01-0627

[B9] Chang H. Shi C. Li M. Zhang T. Wang C. Jiang L. (2018). The effect of cations (NH4+, Na+, K+, and Ca2+) on chemical deactivation of commercial SCR catalyst by bromides. Chin. J. Catal. 39, 710–717. 10.1016/S1872-2067(18)63011-6

[B10] Chen X. Geng Y. Shan W. Liu F. (2018). Deactivation effects of potassium on a CeMoTiO_x_ catalyst for the selective catalytic reduction of NO_x_ with NH_3_ . Ind. Eng. Chem. Res. 57, 1399–1407. 10.1021/acs.iecr.7b04444

[B11] Damma D. Ettireddy P. R. Reddy B. M. Smirniotis P. G. (2019). A review of low temperature NH3-SCR for removal of NOx. Catalysts 9, 349. 10.3390/catal9040349

[B12] Du J. Shi X. Shan Y. Xu G. Sun Y. Wang Y. (2020). Effects of SO 2 on Cu-SSZ-39 catalyst for the selective catalytic reduction of NO x with NH 3. Catal. Sci. Technol. 10, 1256–1263. 10.1039/C9CY02186H

[B13] Du M. Niu Y. Hu B. Zhou G. Luo H. Qi X. (2021). Frequency regulation analysis of modern power systems using start-stop peak shaving and deep peak shaving under different wind power penetrations. Int. J. Electr. Power & Energy Syst. 125, 106501. 10.1016/j.ijepes.2020.106501

[B14] Fan J. Ning P. Song Z. Liu X. Wang L. Wang J. (2018). Mechanistic aspects of NH3-SCR reaction over CeO2/TiO2-ZrO2-SO42− catalyst: *In situ* DRIFTS investigation. Chem. Eng. J. 334, 855–863. 10.1016/j.cej.2017.10.011

[B15] Fu-Dong L. Wen-Po D. Xiao-Yan S. Chang-Bin Z. Hong H. (2011). Research progress in vanadium-free catalysts for the selective catalytic reduction of NO with NH3. Chin. J. Catal. 32, 1113–1128. 10.3724/SP.J.1088.2011.10315

[B16] Gao F. Mei D. Wang Y. Szanyi J. Peden C. H. (2017). Selective catalytic reduction over Cu/SSZ-13: Linking homo-and heterogeneous catalysis. J. Am. Chem. Soc. 139, 4935–4942. 10.1021/jacs.7b01128 28288511

[B17] Gao F. Tang X. Sani Z. Yi H. Zhao S. Yu Q. (2020). Spinel-structured Mn–Ni nanosheets for NH 3-SCR of NO with good H 2 O and SO 2 resistance at low temperature. Catal. Sci. Technol. 10, 7486–7501. 10.1039/D0CY01337D

[B18] Gao X. Du X.-S. Cui L.-W. Fu Y.-C. Luo Z.-Y. Cen K.-F. (2010a). A Ce–Cu–Ti oxide catalyst for the selective catalytic reduction of NO with NH3. Catal. Commun. 12, 255–258. 10.1016/j.catcom.2010.09.029

[B19] Gao X. Jiang Y. Zhong Y. Luo Z. Cen K. (2010b). The activity and characterization of CeO2-TiO2 catalysts prepared by the sol–gel method for selective catalytic reduction of NO with NH3. J. Hazard. Mater. 174, 734–739. 10.1016/j.jhazmat.2009.09.112 19837510

[B20] Gu Y. Xu J. Chen D. Wang Z. Li Q. (2016). Overall review of peak shaving for coal-fired power units in China. Renew. Sustain. Energy Rev. 54, 723–731. 10.1016/j.rser.2015.10.052

[B21] Guo M. Lis B. M. Ford M. E. Wachs I. E. (2022). The effect of non-redox promoters (AlOx, POx, SiOx and ZrOx) and surface sulfates on supported V2O5-WO3/TiO2 catalysts in selective catalytic reduction of NO with NH3. Appl. Catal. B Environ. 121128. 10.1016/j.apcatb.2022.121128

[B22] Guo X. Bartholomew C. Hecker W. Baxter L. L. (2009). Effects of sulfate species on V2O5/TiO2 SCR catalysts in coal and biomass-fired systems. Appl. Catal. B Environ. 92, 30–40. 10.1016/j.apcatb.2009.07.025

[B23] Han J. Meeprasert J. Maitarad P. Nammuangruk S. Shi L. Zhang D. (2016). Investigation of the facet-dependent catalytic performance of Fe2O3/CeO2 for the selective catalytic reduction of NO with NH3. J. Phys. Chem. C 120, 1523–1533. 10.1021/acs.jpcc.5b09834

[B24] He J.-F. Xiong Z.-B. Du Y.-P. Lu W. Tian S. l. (2021). Morphology effect of tungsten oxide on Ce/W catalyst for selective catalytic reduction of NO with NH3: Influence of structure-directing agents. J. Energy Inst. 94, 85–95. 10.1016/j.joei.2020.11.003

[B25] Huang J. Tong Z. Huang Y. Zhang J. (2008). Selective catalytic reduction of NO with NH3 at low temperatures over iron and manganese oxides supported on mesoporous silica. Appl. Catal. B Environ. 78, 309–314. 10.1016/j.apcatb.2007.09.031

[B26] Jia Z. Lin B. (2021). How to achieve the first step of the carbon-neutrality 2060 target in China: The coal substitution perspective. Energy 233, 121179. 10.1016/j.energy.2021.121179

[B27] Jiao K. Chen X. Bie X. Liu D. Qiu M. Ma S. (2021). Status and development for detection and control of ammonium bisulfate as a by-product of SCR denitrification. Sci. Rep. 11 (1), 1–13. 10.1038/s41598-021-90040-w 34001981 PMC8129103

[B28] Jin R. Liu Y. Wu Z. Wang H. Gu T. (2010). Relationship between SO2 poisoning effects and reaction temperature for selective catalytic reduction of NO over Mn–Ce/TiO2 catalyst. Catal. Today 153, 84–89. 10.1016/j.cattod.2010.01.039

[B29] Kang M. Park E. D. Kim J. M. Yie J. E. (2007). Manganese oxide catalysts for NOx reduction with NH3 at low temperatures. Appl. Catal. A general 327, 261–269. 10.1016/j.apcata.2007.05.024

[B30] Kang M. Yeon T. H. Park E. D. Yie J. E. Kim J. M. (2006). Novel MnO x catalysts for NO reduction at low temperature with ammonia. Catal. Lett. 106, 77–80. 10.1007/s10562-005-9194-3

[B31] Kapteijn F. Singoredjo L. Andreini A. Moulijn J. (1994). Activity and selectivity of pure manganese oxides in the selective catalytic reduction of nitric oxide with ammonia. Appl. Catal. B Environ. 3, 173–189. 10.1016/0926-3373(93)E0034-9

[B32] Kim S. S. Choi S. H. Lee S. M. Hong S. C. (2012). Enhanced catalytic activity of Pt/Al2O3 on the CH4 SCR. J. Industrial Eng. Chem. 18, 272–276. 10.1016/j.jiec.2011.11.041

[B33] Koebel M. Elsener M. Madia G. (2001). Reaction pathways in the selective catalytic reduction process with NO and NO2 at low temperatures. Ind. Eng. Chem. Res. 40, 52–59. 10.1021/ie000551y

[B34] Kröcher O. Brandenberger S. (2012). Active sites, deactivation and stabilization of Fe-ZSM-5 for the selective catalytic reduction (SCR) of NO with NH3. Chim. (Aarau). 66, 687–693. 10.2533/chimia.2012.687 23211727

[B35] Lee K. Choi B. Kim C. Lee C. Oh K. (2021). De-NOx characteristics of HC-SCR system employing combined Ag/Al2O3 and CuSn/ZSM-5 catalyst. J. Industrial Eng. Chem. 93, 461–475. 10.1016/j.jiec.2020.10.026

[B36] Li J. Peng Y. Chang H. Li X. Crittenden J. C. Hao J. (2016). Chemical poison and regeneration of SCR catalysts for NO x removal from stationary sources. Front. Environ. Sci. Eng. 10, 413–427. 10.1007/s11783-016-0832-3

[B37] Li J. Tang X. Gao F. Yi H. Zhao S. (2017). Studies on the calcium poisoning and regeneration of commercial De-NO x SCR catalyst. Chem. Pap. 71, 1921–1928. 10.1007/s11696-017-0186-8

[B38] Li J. Zhang P. Chen L. Zhang Y. Qi L. (2020). Regeneration of selective catalyst reduction catalysts deactivated by Pb, as, and alkali metals. ACS omega 5, 13886–13893. 10.1021/acsomega.0c01283 32566855 PMC7301543

[B39] Li P. Xin Y. Li Q. Wang Z. Zhang Z. Zheng L. (2012). Ce–Ti amorphous oxides for selective catalytic reduction of NO with NH3: Confirmation of Ce–O–Ti active sites. Environ. Sci. Technol. 46, 9600–9605. 10.1021/es301661r 22888951

[B40] Li Y. Z. Q. (2009). Recent advances in mechanisms and kinetics of low-temperature selective catalytic reduction of NOx with NH3. Prog. Chem. 21, 1094. WOS:000267348400004.

[B41] Lin L. Xu B. Xia S. (2019). Multi-angle economic analysis of coal-fired units with plasma ignition and oil injection during deep peak shaving in China. Appl. Sci. 9, 5399. 10.3390/app9245399

[B42] Lin Q.-C. Hao J.-M. Li J.-H. (2006). Fe promotion effect in Mn/USY for low-temperature selective catalytic reduction of NO with NH3. Chin. Chem. Lett. 17, 991–994. CNKI:SUN:FXKB.0.2006-07-038.

[B43] Lisi L. Cimino S. (2020). Poisoning of SCR catalysts by alkali and alkaline earth metals. Catalysts 10, 1475. 10.3390/catal10121475

[B44] Liu C. Wang H. Zhang Z. Liu Q. (2020). The latest research progress of NH3-SCR in the SO2 resistance of the catalyst in low temperatures for selective catalytic reduction of NOx. Catalysts 10, 1034. 10.3390/catal10091034

[B45] Liu F. Shan W. Lian Z. Liu J. He H. (2018a). The smart surface modification of Fe2O3 by WO for significantly promoting the selective catalytic reduction of NO with NH3. Appl. Catal. B Environ. 230, 165–176. 10.1016/j.apcatb.2018.02.052

[B46] Liu J. Meeprasert J. Namuangruk S. Zha K. Li H. Huang L. (2017). Facet–activity relationship of TiO2 in Fe2O3/TiO2 nanocatalysts for selective catalytic reduction of NO with NH3: *In situ* DRIFTs and DFT studies. J. Phys. Chem. C 121, 4970–4979. 10.1021/acs.jpcc.6b11175

[B47] Liu J. Wei Y. Li P.-Z. Zhang P. Su W. Sun Y. (2018b). Experimental and theoretical investigation of mesoporous MnO_2_ nanosheets with oxygen vacancies for high-efficiency catalytic DeNO_x_ . ACS Catal. 8, 3865–3874. 10.1021/acscatal.8b00267

[B48] Liu Z. Liu H. Feng X. Ma L. Cao X. Wang B. (2018c). Ni-Ce-Ti as a superior catalyst for the selective catalytic reduction of NOx with NH3. Mol. Catal. 445, 179–186. 10.1016/j.mcat.2017.11.028

[B49] Liu Z. Su H. Chen B. Li J. Woo S. I. (2016). Activity enhancement of WO3 modified Fe2O3 catalyst for the selective catalytic reduction of NO by NH3. Chem. Eng. J. 299, 255–262. 10.1016/j.cej.2016.04.100

[B50] Liu Z. Zhu J. Zhang S. Ma L. Woo S. I. (2014). Selective catalytic reduction of NOx by NH3 over MoO3-promoted CeO2/TiO2 catalyst. Catal. Commun. 46, 90–93. 10.1016/j.catcom.2013.11.032

[B51] Lu Q. Pei X.-Q. Wu Y.-W. Xu M.-X. Liu D.-J. Zhao L. (2020). Deactivation mechanism of the commercial V2O5–MoO3/TiO2 selective catalytic reduction catalyst by arsenic poisoning in coal-fired power plants. Energy fuels. 34, 4865–4873. 10.1021/acs.energyfuels.0c00066

[B52] Ma H. Zhang Y. Shen M. (2021a). Application and prospect of supercapacitors in internet of energy (IOE). J. Energy Storage 44, 103299. 10.1016/j.est.2021.103299

[B53] Ma S. Gao W. Yang Z. Lin R. Wang X. Zhu X. (2021b). Superior Ce–Nb–Ti oxide catalysts for selective catalytic reduction of NO with NH3. J. Energy Inst. 94, 73–84. 10.1016/j.joei.2020.11.001

[B54] Mallapaty S. (2020). How China could be carbon neutral by mid-century. Nature 586, 482–483. 10.1038/d41586-020-02927-9 33077972

[B55] Miao J. Yi X. Su Q. Li H. Chen J. Wang J. (2020). Poisoning effects of phosphorus, potassium and lead on V2O5-WO3/TiO2 catalysts for selective catalytic reduction with NH3. Catalysts 10, 345. 10.3390/catal10030345

[B56] Ming'e Y. Caiting L. Yan W. Lingling Z. Lei G. (2016). Catalytic performance of the SCR catalyst supported on modified TiO2-SnO2 carrier. Chin. J. Environ. Eng. 10, 3733–3738. 10.12030/j.cjee.201502049

[B57] Mirhashemi F. S. Sadrnia H. (2020). NOX emissions of compression ignition engines fueled with various biodiesel blends: A review. J. Energy Inst. 93, 129–151. 10.1016/j.joei.2019.04.003

[B58] Mladenović M. Paprika M. Marinković A. (2018). Denitrification techniques for biomass combustion. Renew. Sustain. Energy Rev. 82, 3350–3364. 10.1016/j.rser.2017.10.054

[B59] Mou X. Zhang B. Li Y. Yao L. Wei X. Su D. S. (2012). Rod‐shaped Fe2O3 as an efficient catalyst for the selective reduction of nitrogen oxide by ammonia. Angew. Chem. Int. Ed. 51, 2989–2993. 10.1002/anie.201107113 22311597

[B60] Park K. H. Lee S. M. Kim S. S. Kwon D. W. Hong S. C. (2013). Reversibility of Mn valence state in MnOx/TiO2 catalysts for low-temperature selective catalytic reduction for NO with NH3. Catal. Lett. 143, 246–253. 10.1007/s10562-012-0952-8

[B61] Patil S. Vanalakar S. Dhodamani A. Deshmukh S. Patil V. Patil P. (2018). NH3 gas sensing performance of ternary TiO2/SnO2/WO3 hybrid nanostructures prepared by ultrasonic-assisted sol–gel method. J. Mat. Sci. Mat. Electron. 29, 11830–11839. 10.1007/s10854-018-9283-x

[B62] Peña D. A. Uphade B. S. Smirniotis P. G. (2004). TiO2-supported metal oxide catalysts for low-temperature selective catalytic reduction of NO with NH3: I. Evaluation and characterization of first row transition metals. J. Catal. 221, 421–431. 10.1016/j.jcat.2003.09.003

[B63] Peng Y. Li J. Si W. Luo J. Wang Y. Fu J. (2015). Deactivation and regeneration of a commercial SCR catalyst: Comparison with alkali metals and arsenic. Appl. Catal. B Environ. 168, 195–202. 10.1016/j.apcatb.2014.12.005

[B64] Peng Y. Wang D. Li B. Wang C. Li J. Crittenden J. (2017). Impacts of Pb and SO2 poisoning on CeO2–WO3/TiO2–SiO2 SCR catalyst. Environ. Sci. Technol. 51, 11943–11949. 10.1021/acs.est.7b03309 28914048

[B65] Pu Y. Xie X. Jiang W. Yang L. Jiang X. Yao L. (2020). Low-temperature selective catalytic reduction of NOx with NH3 over zeolite catalysts: A review. Chin. Chem. Lett. 31, 2549–2555. 10.1016/j.cclet.2020.04.012

[B66] Qi L. Li J. Yao Y. Zhang Y. (2019). Heavy metal poisoned and regeneration of selective catalytic reduction catalysts. J. Hazard. Mater. 366, 492–500. 10.1016/j.jhazmat.2018.11.112 30562661

[B67] Qiu L. Meng J. Pang D. Zhang C. Ouyang F. (2015). Reaction and characterization of Co and Ce doped Mn/TiO2 catalysts for low-temperature SCR of NO with NH3. Catal. Lett. 145, 1500–1509. 10.1007/s10562-015-1556-x

[B68] Qu R. Gao X. Cen K. Li J. (2013). Relationship between structure and performance of a novel cerium-niobium binary oxide catalyst for selective catalytic reduction of NO with NH3. Appl. Catal. B Environ. 142, 290–297. 10.1016/j.apcatb.2013.05.035

[B69] Ren Z. Fan H. Wang R. (2017a). A novel ring-like Fe2O3-based catalyst: Tungstophosphoric acid modification, NH3-SCR activity and tolerance to H2O and SO2. Catal. Commun. 100, 71–75. 10.1016/j.catcom.2017.06.038

[B70] Ren Z. Teng Y. Zhao L. Wang R. (2017b). Keggin-tungstophosphoric acid decorated Fe2O3 nanoring as a new catalyst for selective catalytic reduction of NOx with ammonia. Catal. Today 297, 36–45. 10.1016/j.cattod.2017.06.036

[B71] Ruliang N. Xiaolong L. Tingyu Z. (2019). Research progress of low-temperature SCR denitration catalysts. Chin. J. Process Eng. 19 (2), 223. Available at: http://ir.ipe.ac.cn/handle/122111/46291 .

[B72] Ryu T. Kim H. Hong S. B. (2019). Nature of active sites in Cu-LTA NH3-SCR catalysts: A comparative study with Cu-SSZ-13. Appl. Catal. B Environ. 245, 513–521. 10.1016/j.apcatb.2019.01.006

[B73] Sakurai S. Namai A. Hashimoto K. Ohkoshi S.-I. (2009). First observation of phase transformation of all four Fe2O3 phases (γ→ ε→ β→ α-phase). J. Am. Chem. Soc. 131, 18299–18303. 10.1021/ja9046069 19938830

[B74] Shan Y. Shan W. Shi X. Du J. Yu Y. He H. (2020). A comparative study of the activity and hydrothermal stability of Al-rich Cu-SSZ-39 and Cu-SSZ-13. Appl. Catal. B Environ. 264, 118511. 10.1016/j.apcatb.2019.118511

[B75] Shen M. Ai F. Ma H. Xu H. Zhang Y. (2021a). Progress and prospects of reversible solid oxide fuel cell materials. Iscience 24, 103464. 10.1016/j.isci.2021.103464 34934912 PMC8661483

[B76] Shen M. Zhang P. (2020). Progress and challenges of cathode contact layer for solid oxide fuel cell. Int. J. Hydrogen Energy 45, 33876–33894. 10.1016/j.ijhydene.2020.09.147

[B77] Shen M. Zhang Y. Xu H. Ma H. (2021b). MOFs based on the application and challenges of perovskite solar cells. Iscience 24, 103069. 10.1016/j.isci.2021.103069 34568791 PMC8449091

[B78] Sheng Z. Yufeng H. Jianming X. Xiaoming W. Weiping L. (2012). SO2 poisoning and regeneration of Mn-Ce/TiO2 catalyst for low temperature NOx reduction with NH3. J. Rare Earths 30, 676–682. 10.1016/S1002-0721(12)60111-2

[B79] Sönmezoğlu S. Arslan A. Serin T. Serin N. (2011). The effects of film thickness on the optical properties of TiO2–SnO2 compound thin films. Phys. Scr. 84, 065602. 10.1088/0031-8949/84/06/065602

[B80] Sun Q. Gao Z.-X. Chen H.-Y. Sachtler W. M. (2001). Reduction of NOx with ammonia over Fe/MFI: Reaction mechanism based on isotopic labeling. J. Catal. 201, 89–99. 10.1006/jcat.2001.3228

[B81] Tang X. Hao J. Xu W. Li J. (2007). Low temperature selective catalytic reduction of NO with NH3 over amorphous MnO catalysts prepared by three methods. Catal. Commun. 8, 329–334. 10.1016/j.catcom.2006.06.025

[B82] Tang X. Li J. Sun L. Hao J. (2010). Origination of N2O from NO reduction by NH3 over β-MnO2 and α-Mn2O3. Appl. Catal. B Environ. 99, 156–162. 10.1016/j.apcatb.2010.06.012

[B83] Tran T.-S. Yu J. Li C. Guo F. Zhang Y. Xu G. (2017). Structure and performance of a V 2 O 5–WO 3/TiO 2–SiO 2 catalyst derived from blast furnace slag (BFS) for DeNO x. RSC Adv. 7, 18108–18119. 10.1039/C7RA01252G

[B84] Wang J. Zhang S. Huo J. Zhou Y. Li L. Han T. (2021). Dispatch optimization of thermal power unit flexibility transformation under the deep peak shaving demand based on invasive weed optimization. J. Clean. Prod. 315, 128047. 10.1016/j.jclepro.2021.128047

[B85] Wang J. Zhao H. Haller G. Li Y. (2017). Recent advances in the selective catalytic reduction of NOx with NH3 on Cu-Chabazite catalysts. Appl. Catal. B Environ. 202, 346–354. 10.1016/j.apcatb.2016.09.024

[B86] Wang L. Wang X. Cheng J. Ning P. Lin Y. (2018). Coupling catalytic hydrolysis and oxidation on Mn/TiO2-Al2O3 for HCN removal. Appl. Surf. Sci. 439, 213–221. 10.1016/j.apsusc.2018.01.015

[B87] Wang X. Gui K. (2013). Fe2O3 particles as superior catalysts for low temperature selective catalytic reduction of NO with NH3. J. Environ. Sci. 25, 2469–2475. 10.1016/S1001-0742(12)60331-3 24649679

[B88] Williams J. H. Jones R. A. Haley B. Kwok G. Hargreaves J. Farbes J. (2021). Carbon-neutral pathways for the United States. AGU Adv. 2, e2020AV000284. 10.1029/2020AV000284

[B89] Wu R. Li L. Zhang N. He J. Song L. Zhang G. (2021). Enhancement of low-temperature NH3-SCR catalytic activity and H2O & SO2 resistance over commercial V2O5-MoO3/TiO2 catalyst by high shear-induced doping of expanded graphite. Catal. Today 376, 302–310. 10.1016/j.cattod.2020.04.051

[B90] Wu Z. Jiang B. Liu Y. (2008). Effect of transition metals addition on the catalyst of manganese/titania for low-temperature selective catalytic reduction of nitric oxide with ammonia. Appl. Catal. B Environ. 79, 347–355. 10.1016/j.apcatb.2007.09.039

[B91] Xie G. Liu Z. Zhu Z. Liu Q. Ma J. (2003). Reductive regeneration of sulfated CuO/Al2O3 catalyst-sorbent in ammonia. Appl. Catal. B Environ. 45, 213–221. 10.1016/S0926-3373(03)00166-8

[B92] Xin Y. Zhang N. Li Q. Zhang Z. Cao X. Zheng L. (2018a). Active site identification and modification of electronic states by atomic-scale doping to enhance oxide catalyst innovation. ACS Catal. 8, 1399–1404. 10.1021/acscatal.7b02638

[B93] Xin Y. Zhang N. Li Q. Zhang Z. Cao X. Zheng L. (2018b). Selective catalytic reduction of NO with NH3 over short-range ordered W O Fe structures with high thermal stability. Appl. Catal. B Environ. 229, 81–87. 10.1016/j.apcatb.2018.02.012

[B94] Xu D. Wu W. Wang P. Deng J. Yan T. Zhang D. (2020a). Boosting the alkali/heavy metal poisoning resistance for NO removal by using iron-titanium pillared montmorillonite catalysts. J. Hazard. Mater. 399, 122947. 10.1016/j.jhazmat.2020.122947 32521318

[B95] Xu H. Shen M. (2021). The control of lithium‐ion batteries and supercapacitors in hybrid energy storage systems for electric vehicles: A review. Intl. J. Energy Res. 45, 20524–20544. 10.1002/er.7150

[B96] Xu J. Wang H. Guo F. Zhang C. Xie J. (2019). Recent advances in supported molecular sieve catalysts with wide temperature range for selective catalytic reduction of NO X with C 3 H 6. RSC Adv. 9, 824–838. 10.1039/C8RA08635D 35517600 PMC9059638

[B97] Xu L. Wu Q. Chang H. Li G. Zou J. Wang S. (2020b). Chemical deactivation of Selective Catalytic Reduction catalyst: Investigating the influence and mechanism of SeO2 poisoning. Fuel 269, 117435. 10.1016/j.fuel.2020.117435

[B98] Xu W. Yu Y. Zhang C. He H. (2008). Selective catalytic reduction of NO by NH3 over a Ce/TiO2 catalyst. Catal. Commun. 9, 1453–1457. 10.1016/j.catcom.2007.12.012

[B99] Xue Y. Ge Z. Yang L. Du X. (2019). Peak shaving performance of coal-fired power generating unit integrated with multi-effect distillation seawater desalination. Appl. Energy 250, 175–184. 10.1016/j.apenergy.2019.04.190

[B100] Yan H. Qu H. Bai H. Zhong Q. (2015). Property, active species and reaction mechanism of NO and NH3 over mesoporous Fe-Al-SBA-15 via microwave assisted synthesis for NH3-SCR. J. Mol. Catal. A Chem. 403, 1–9. 10.1016/j.molcata.2015.02.018

[B101] Yan L. Gu Y. Han L. Wang P. Li H. Yan T. (2019). Dual promotional effects of TiO_2_-decorated acid-treated MnO_x_ octahedral molecular sieve catalysts for alkali-resistant reduction of NO_x_ . ACS Appl. Mat. Interfaces 11, 11507–11517. 10.1021/acsami.9b01291 30817117

[B102] Yan R. Lin S. Li Y. Liu W. Mi Y. Tang C. (2020). Novel shielding and synergy effects of Mn-Ce oxides confined in mesoporous zeolite for low temperature selective catalytic reduction of NOx with enhanced SO2/H2O tolerance. J. Hazard. Mater. 396, 122592. 10.1016/j.jhazmat.2020.122592 32298863

[B103] Yang W. Liu F. Xie L. Lian Z. He H. (2016). Effect of V_2_O_5_ additive on the SO_2_ resistance of a Fe_2_O_3_/AC catalyst for NH_3_-SCR of NO_x_ at low temperatures. Ind. Eng. Chem. Res. 55, 2677–2685. 10.1021/acs.iecr.5b04974

[B104] Yu Y. He C. Chen J. Yin L. Qiu T. Meng X. (2013). Regeneration of deactivated commercial SCR catalyst by alkali washing. Catal. Commun. 39, 78–81. 10.1016/j.catcom.2013.05.010

[B105] Yu Y. Tan W. An D. Wang X. Liu A. Zou W. (2021). Insight into the SO2 resistance mechanism on γ-Fe2O3 catalyst in NH3-SCR reaction: A collaborated experimental and dft study. Appl. Catal. B Environ. 281, 119544. 10.1016/j.apcatb.2020.119544

[B106] Zeng Z. Lu P. Li C. Zeng G. Jiang X. Zhai Y. (2012). Selective catalytic reduction (SCR) of NO by urea loaded on activated carbon fibre (ACF) and CeO2/ACF at 30 C: the SCR mechanism. Environ. Technol. 33, 1331–1337. 10.1080/09593330.2011.626799 22856306

[B107] Zhang M. Gu K. Huang X. Chen Y. (2019a). A DFT study on the effect of oxygen vacancies and H 2 O in Mn-MOF-74 on SCR reactions. Phys. Chem. Chem. Phys. 21, 19226–19233. 10.1039/C9CP02640A 31441492

[B108] Zhang N. Xin Y. Wang X. Shao M. Li Q. Ma X. (2017). Iron-niobium composite oxides for selective catalytic reduction of NO with NH3. Catal. Commun. 97, 111–115. 10.1016/j.catcom.2017.04.033

[B109] Zhang Q. Wu Y. Yuan H. (2020). Recycling strategies of spent V2O5-WO3/TiO2 catalyst: A review. Resour. Conservation Recycl. 161, 104983. 10.1016/j.resconrec.2020.104983

[B110] Zhang R. Liu N. Lei Z. Chen B. (2016). Selective transformation of various nitrogen-containing exhaust gases toward N2 over zeolite catalysts. Chem. Rev. 116, 3658–3721. 10.1021/acs.chemrev.5b00474 26889565

[B111] Zhang Y. Xin X. Hu D. Zhu W. Zhu Y. (2019b). Study on stability and reliability of NOx ultra-low emission in coal-fired power plants in China. E3S Web of Conferences. EDP Sciences, 03002. 10.1051/e3sconf/201912003002

[B112] Zhang Z. Li J. Tian J. Zhong Y. Zou Z. Dong R. (2022). The effects of Mn-based catalysts on the selective catalytic reduction of NOx with NH3 at low temperature: A review. Fuel Process. Technol. 230, 107213. 10.1016/j.fuproc.2022.107213

[B113] Zhao G. Li M. Wang L. Wang D. Liang J. Xue G. (2020). Environmentally-friendly tourmaline modified CeMnFeOx catalysts for low-temperature selective catalytic reduction of NOx with NH3. Catal. Today 355, 385–396. 10.1016/j.cattod.2019.08.018

[B114] Zhe L. Shen L.-T. Huang W. Xie K.-C. (2007). Kinetics of selective catalytic reduction of NO by NH3 on Fe-Mo/ZSM-5 catalyst. J. Environ. Sci. 19, 1516–1519. 10.1016/S1001-0742(07)60247-2 18277659

[B115] Zheng L. Casapu M. Stehle M. Deutschmann O. Grunwaldt J.-D. (2019). Selective catalytic reduction of NOx with ammonia and hydrocarbon oxidation over V2O5–MoO3/TiO2 and V2O5–WO3/TiO2 SCR catalysts. Top. Catal. 62, 129–139. 10.1007/s11244-018-1097-9

[B116] Zheng Y. Jensen A. D. Johnsson J. E. (2004). Laboratory investigation of selective catalytic reduction catalysts: Deactivation by potassium compounds and catalyst regeneration. Ind. Eng. Chem. Res. 43, 941–947. 10.1021/ie030404a

[B117] Zhigang L. Aibin L. Meiru J. Xueyi L. (2010). Experimental and kinetic study of selective catalytic reduction of NO with NH3 over CuO/Al2O3/Cordierite catalyst. Chin. J. Chem. Eng. 18, 721–729. 10.1016/S1004-9541(09)60120-8

